# Transcriptional profiling provides new insights into the role of nitric oxide in enhancing *Ganoderma oregonense* resistance to heat stress

**DOI:** 10.1038/s41598-017-15340-6

**Published:** 2017-11-16

**Authors:** Cheng Chen, Qiang Li, Qiangfeng Wang, Daihua Lu, Hong Zhang, Jian Wang, Rongtao Fu

**Affiliations:** 1 Institute of plant protection, Sichuan Academy of Agricultural Sciences, Chengdu, 610066 P.R. China; 2 Biotechnology and Nuclear Technology Research Institute, Sichuan Academy of Agricultural Sciences, Chengdu, 610061 Sichuan P.R. China; 3 Key Laboratory of Bio-Resource and Eco-Environment of Ministry of Education, College of Life Sciences, Sichuan University, Chengdu, 610065 Sichuan P.R. China; 4 Present Address: Sichuan Academy of Agricultural Sciences, 20 # Jingjusi Rd, Chengdu, 610066 Sichuan China

## Abstract

*Ganoderma* is well known for its use in traditional Chinese medicine and is widely cultivated in China, Korea, and Japan. Increased temperatures associated with global warming are negatively influencing the growth and development of *Ganoderma*. Nitric oxide is reported to play an important role in alleviating fungal heat stress (HS). However, the transcriptional profiling of *Ganoderma oregonense* in response to HS, as well as the transcriptional response regulated by NO to cope with HS has not been reported. We used RNA-Seq technology to generate large-scale transcriptome data from *G. oregonense* mycelia subjected to HS (32 °C) and exposed to concentrations of exogenous NO. The results showed that heat shock proteins (HSPs), “probable stress-induced proteins”, and unigenes involved in “D-amino-acid oxidase activity” and “oxidoreductase activity” were significantly up-regulated in *G. oregonense* subjected to HS (*P* < 0.05). The significantly up-regulated HSPs, “monooxygenases”, “alcohol dehydrogenase”, and “FAD/NAD(P)-binding domain-containing proteins” (*P* < 0.05) regulated by exogenous NO may play important roles in the enhanced HS tolerance of *G. oregonense*. These results provide insights into the transcriptional response of *G. oregonense* to HS and the mechanism by which NO enhances the HS tolerance of fungi at the gene expression level.

## Introduction

*Ganoderma* spp. is a group of white rot fungus belonging to the Ganodermataceae, Polyporales^1–3^. The medicinal effects of the *Ganoderma* complex have been intensively studied. Triterpenes, polysaccharide peptides, polysaccharides, ganoderic acids, and ribonucleases isolated from the fruiting bodies and fermentation of *Ganoderma* spp. exhibit antioxidant, antiallergic, antiinflammatory, antifungal, antitumor, immunostimulatory, and mitogenic activities^4–12^. As a species of *G. lucidum* complex, *G. oregonense* was first discovered in North America^1^. And this species was successfully introduced from the United States through cooperation and cultivated in southwestern China, which supported by a *Ganoderma* sp. breeding project. This species is associated with good agronomic traits, including a high biological conversion rate, a large cap, and high yield. It has the potential for further cultivation in both China and other parts of the world. Investigating the response of this species to stress should provide information for the successful cultivation of *G. oregonense* as well as other *Ganoderma* species under conditions of abiotic stress.

Organisms are affected by environmental changes, and excessively high temperatures constitute a significant environmental challenge. Microorganisms are directly exposed to the temperature alterations associated with climate change since they cannot move to avoid high temperature stress. Heat stress (HS) is often reported to affect the physiological processes and growth of microorganisms. The survival rate of *Saccharomyces cerevisiae* and fatty acid synthesis of *Candida albicans* were seriously affected by HS^13–15^. HS is one of the important environmental factors negatively affecting the growth and development of edible fungi^16^. The pileus diameter and biomass of the *Agaricus bisporus* fruiting body was severely reduced after several days of exposure to high temperatures^17^. HS can also induce apoptotic-like cell death in *Pleurotus* species and inhibit mycelium growth, impair fruiting, and affect mushroom quality^18^. To cope with HS, several physiological changes, such as the production of DNA methyltransferases, DNA-binding proteins, compatible solute accumulation, expression of heat shock proteins (HSPs), activation of reactive oxygen species (ROS) scavenging enzymes, and alterations in morphology induced by microorganisms play important roles in the heat stress response (HSR)^19–21^. Transcriptome or gene expression profiles reveal that some genes related to biological regulation, cellular processes, and metabolic processes are involved in HSR^22^. The yields of some secondary metabolites, such as ganoderic acids (GA) and triterpenes, were increased in *Ganoderma* sp. under HS, which indicates that *Ganoderma* sp. can be used as a model system for evaluating how environmental factors regulate the development and secondary metabolism of basidiomycetes^23,24^. However, the transcriptional profiles and genes related to the response of *G. oregonense* to HS are not yet known.

Nitric oxide (NO) is a simple, small gas molecule that can easily diffuse through cell membranes. It plays an important role in the growth and development of organisms^25^. NO regulates the morphogenesis, sporulation, spore germination, cellular development, apoptosis, and reproduction of fungi. NO also plays an important role in the fungal response to abiotic stress^26,27^. Kong *et al*. reported that NO could alleviate heat stress through the regulation of trehalose accumulation and reactive oxygen species (ROS)-scavenging enzymes in *Pleurotus eryngii* ^28^. The tolerance of *Ganoderma lucidum* to cadmium was also enhanced by NO^29^. However, the transcriptional response of fungi to the abiotic stress regulated by NO, especially to heat stress, is still unknown. Since well-grown hyphae constitute the basis for successful cultivation, and the associated conditions are easier to control in the laboratory, we therefore selected to use the hyphae to investigate the NO-regulated response to heat stress. In this study, we used high-throughput RNA-Seq technology to study the transcriptional responses of *G. oregonense* to heat stress and the mechanism by which NO enhances fungal resistance to HS at the gene expression level. The effect of exogenous NO on hyphal gene expression under non-stress conditions was also assessed. This is the first report on the transcriptional profiling of *G. oregonense* in response to heat stress, as well as the transcriptional responses regulated by NO to cope with abiotic stress. This research illustrates the role of NO in enhancing fungal resistance to abiotic stress at the gene expression level and provides a potential application for the cultivation of fungi under abiotic stress.

## Results

### Effect of SNP on fresh biomass

**Figure 1:**
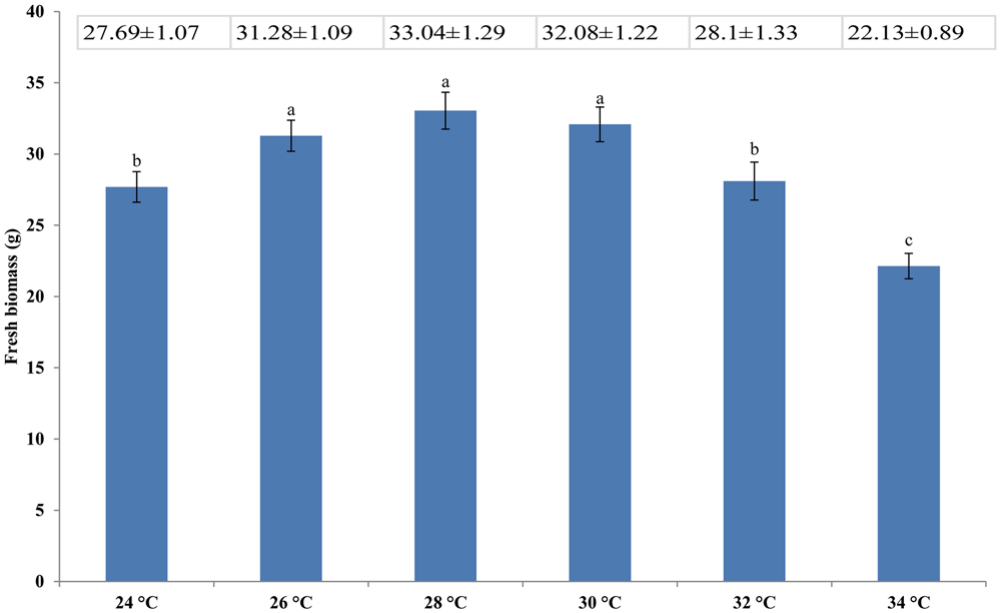
Effect of different temperatures on the fresh biomass of *G. oregonense*. Vertical bars represent the SD of the mean (n = 3). Means with different letter (s) are significantly different (*P* < 0.05).

**Figure 2:**
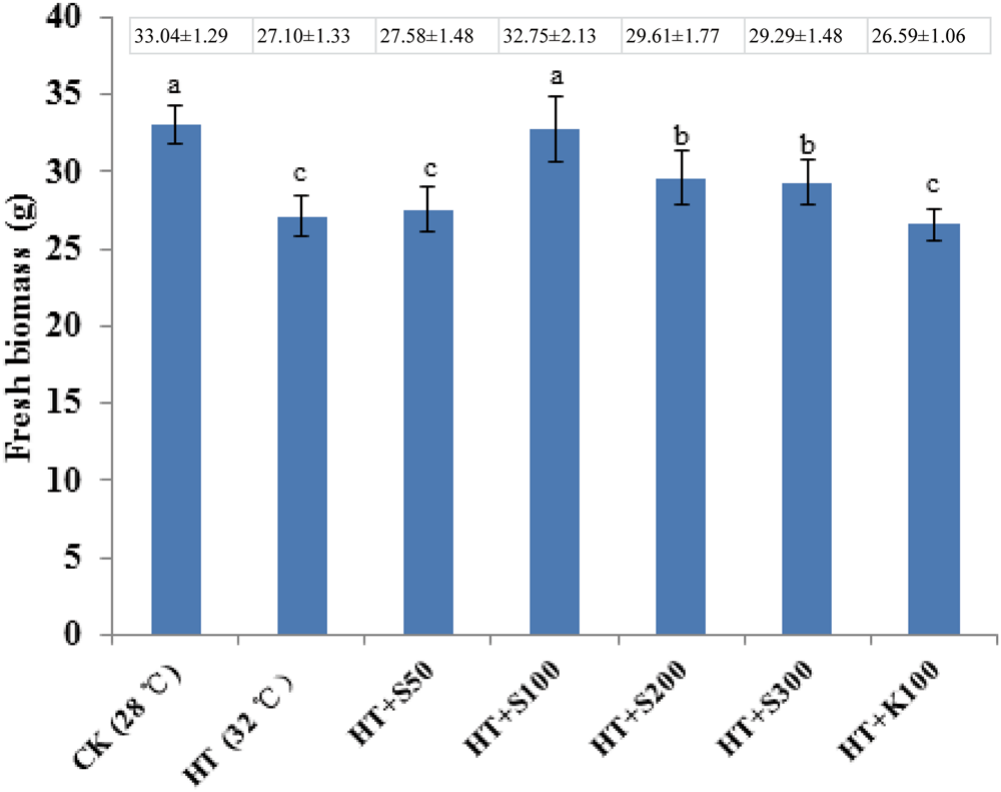
Effect of SNP on the fresh biomass of *G. oregonense* suffering from heat stress. CK, *G. oregonense* mycelia treated at 28°C; HT, *G. oregonense* mycelia treated at 32 °C; HT + S50, *G. oregonense* mycelia treated at 32 °C with 50 µM SNP; HT + S100, *G. oregonense* mycelia treated at 32°C with 100 µM SNP; HT + S200, *G. oregonense* mycelia treated at 32°C with 200 µM SNP; HT + S300, *G. oregonense* mycelia treated at 32°C with 300 µM SNP; HT + K100, *G. oregonense* mycelia treated at 32°C with 150 µM K4[Fe(CN)6]. Vertical bars represent the SD of the mean (n = 3). Means with different letter (s) are significantly different (*P* < 0.05).

The biomass of *G. oregonense* treated at 28 °C was the highest among all treatments (Fig. 1). It was significantly decreased when treated at 32 °C, indicating that the mycelia were heat stressed at 32 °C. At 34 °C, the mycelial growth was severely inhibited, and some hyphae were destroyed. We therefore selected to use 32 °C in the heat treatment because this temperature significantly inhibited the growth of hyphae, resulting in slow growth, but it did not kill them. Mycelia treated at 28 °C were therefore set as the control (CK). Compared with the CK, 32 °C resulted in a sharp decrease in the fresh biomass of *G. oregonense*. However, there was a significant increase in fresh biomass in the 32 °C treatment in combination with 100 µM SNP (Fig. 2). Significant growth inhibition (*P* < 0.05) occurred at 32 °C with K4 [Fe(CN)6], which was similar to treatment at 32 °C alone. Mycelia treated at 32 °C revealed the transcriptional responses of *G. oregonense* to heat stress. Samples treated at 32 °C with 100 µM nitric oxide donor revealed the mechanism by which NO enhances fungal tolerance to abiotic stress. The mycelia treated at 28 °C were assigned to CK, the mycelia treated at 32 °C were assigned to HT, the mycelia treated at 28 °C with 100 µM SNP were assigned to CKSNP, and the mycelia treated at 32 °C with 100 µM SNP were assigned to HTSNP.

### Sequencing of the *G. oregonense* transcriptome and de novo assembly

To study the transcriptional responses of *G. oregonense* to heat stress and the mechanism of NO–induced enhancement of fungal resistance to abiotic stress, *G. oregonense* mycelia treated at 28 °C, 32 °C, and at 28 °C or 32 °C with an NO donor (100 µM SNP) were subjected to RNA-Seq analysis on the Illumina HiSeq 2000 platform. A total of 3.70 × 10^8^ clean reads from 3.74 × 10^8^ raw reads, and 5.43 × 10^10^ clean bases from 5.62 × 10^10^ raw bases were generated (Table S1). Approximately 1.07% of the low quality reads were discarded from the raw data. All the downstream analyses were based on high quality, clean data. The Q20, Q30, and GC-contents of the clean data were calculated (Table S1). These high-quality reads were assembled into 186,159 contigs, 110,631 transcripts, and 58,550 unigenes. The contig length, transcript length, and unigene length distribution are shown in Table S2. The maximum contig length was 15,441 bp, with a mean length of 348 bp. Transcripts less than 500 bp in length occupied the largest proportion of all the assembled transcripts, with a mean length of 1,107 bp. More than half of the unigenes were between 200 and 400 bp long, with a mean unigene length of 767 bp.

### Functional annotation and characterization of *G. oregonense* transcripts

The entire set of unigenes was annotated in five frequently used databases, including the NCBI non-redundant (Nr) protein, the evolutionary genealogy of genes: Non-supervised Orthologous Groups (eggNOG), the SwissProt protein database (SwissProt), the Kyoto Encyclopedia of Genes and Genomes (KEGG), and the Gene Ontology (GO) database. The identity and species distributions were analyzed (Fig. S1). A total of 43.13% unigenes exhibited significant similarity with known proteins in the Nr database. Homology analysis indicated that 57.89% of annotated unigenes had the greatest number of matches with proteins of *Dichomitus squalens* LYAD-421 SS1 (Fig. S2). Additionally, 10.92% of the annotated unigenes were best matched to sequences of *Trametes versicolor* FP-101664 SS1. The remaining 31.18% annotated unigenes had hits with other fungal species, such as *Rhizopus delemar* RA 99–880(2.70%), *Batrachochytrium dendrobatidis* JAM81 (2.63%), *Ceriporiopsis subvermispora* B(1.22%), and *Fibroporia radiculosa* (1.11%). However, 23.52% of the unigenes did not show significant similarity with any other proteins in the Nr database.

### Functional classification

**Figure 3:**
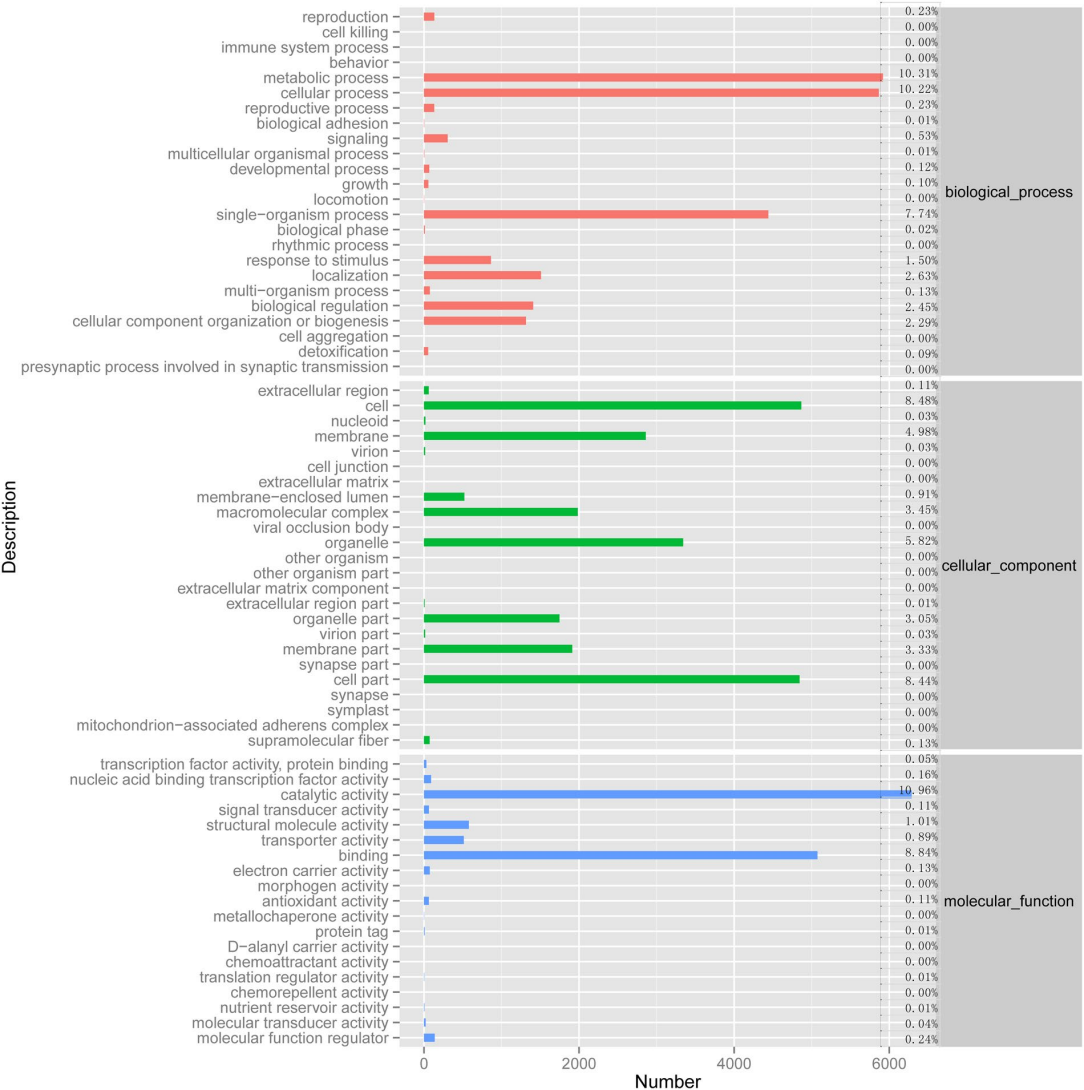
GO classification of the *G. oregonense* transcriptome. The x-axis represents the number of unigenes involved in different GO terms.

GO assignment programs facilitate internationally standardized gene functional categorization. The functions of *G. oregonense* transcripts were classified with GO assignments based on the Nr annotation (Fig. 3). A total of 58,550 unigenes were categorized into 44 functional groups belonging to three GO terms: molecular function, cellular component, and biological process. Under the molecular function category, binding and catalytic activity represented the majority of unigenes. Within the cellular component category, cell parts and cells were dominant. Metabolic process, cellular process, and single-organism process were most highly represented among the biological processes.

**Figure 4:**
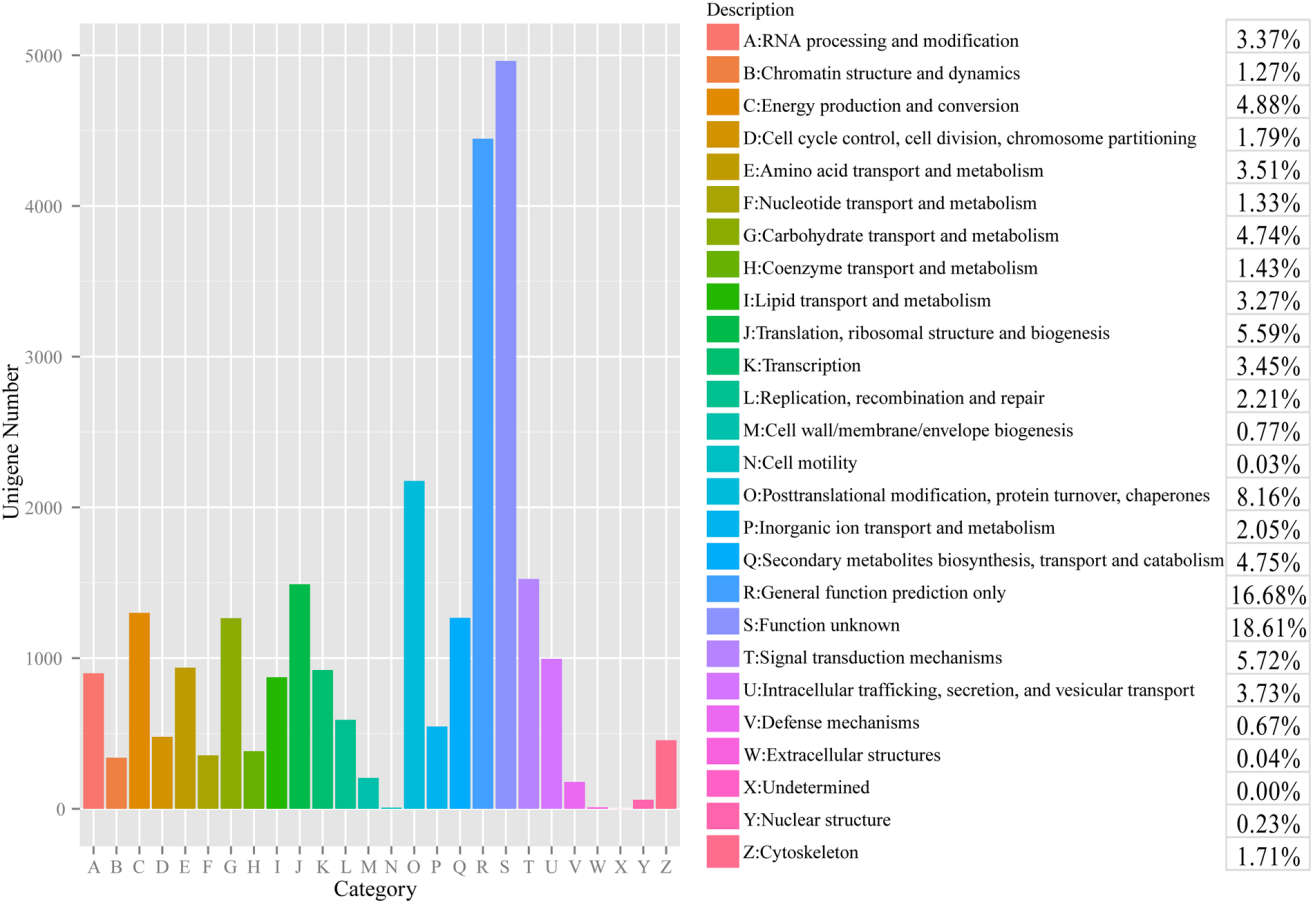
Histogram of the eggNOG classification. A total of 65,064 *G. oregonense* unigenes were assigned to one or more eggNOG functional categories. The letters on the x-axis represent different eggNOG categories. The y-axis represents the number of unigenes participating in different eggNOG categories.

To further evaluate the *G. oregonense* transcriptome and the effectiveness of the annotation process, annotated unigenes were assigned to eggNOG classifications (Fig. 4). Overall, 28,480 unigenes were assigned to one or more eggNOG functional categories. Within the 26 eggNOG categories, the majority of the unigenes were in the “Function unknown” (18.61%) category followed by “General function prediction only” (16.88%), and “Posttranslational modification, protein turnover, chaperones” (8.16%). Few unigenes were assigned to “Extracellular structures” (0.04%) and “Cell motility” (0.03%).

**Figure 5:**
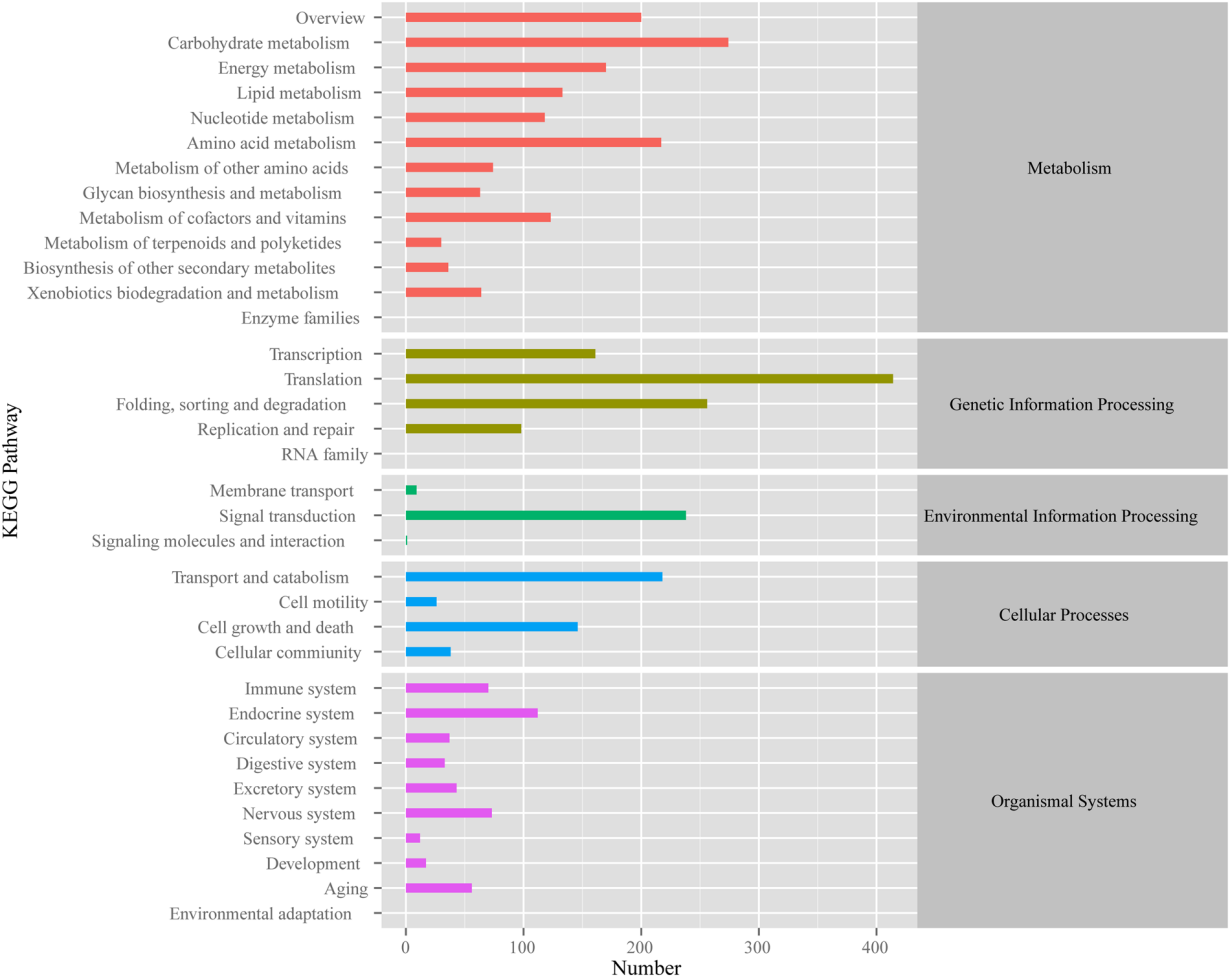
Histogram of the KEGG classification. The letters on the right y-axis represent different KEGG categories: metabolism, genetic information processing, environmental information processing, cellular processes, and organismal systems.

To further elucidate the biological functions and gene interactions of *G. oregonense*, all 58,550 annotated unigenes were mapped to reference biological pathways in the KEGG database (Fig. 5). Approximately 2,870 annotated unigenes had significant matches in the KEGG database to 31 KEGG pathways (*P* < 0.05). Within these unigenes, 1,189 were assigned to metabolism processes in the KEGG database. The pathways containing the most unigenes were involved in “carbohydrate metabolism” (192 unigenes) and “amino acid metabolism” (175 unigenes). In addition, 760 unigenes involved in “translation” (347 unigenes) and “folding, sorting and degradation signal transduction” (203 unigenes) were assigned to the genetic information processing pathway. A total of 374 unigenes were sorted into the organismal systems pathway, containing “endocrine system” (88 unigenes), “nervous system” (69 unigenes), and others. A total of 356 unigenes were classified into cellular processes pathways, including “transport and catabolism” (183 unigenes), “cell growth and death” (115 unigenes), and others. A total of 191 unigenes were involved in the environmental information processing pathway, including “signal transduction” (184 unigenes) and “membrane transport” (seven unigenes). The KEGG pathway annotations provided a valuable resource for investigating the specific processes, functions, and pathways involved in the response of *G. oregonense* to heat stress regulated by NO.

### Differentially expressed genes (DEGs) in response to heat stress

**Figure 6:**
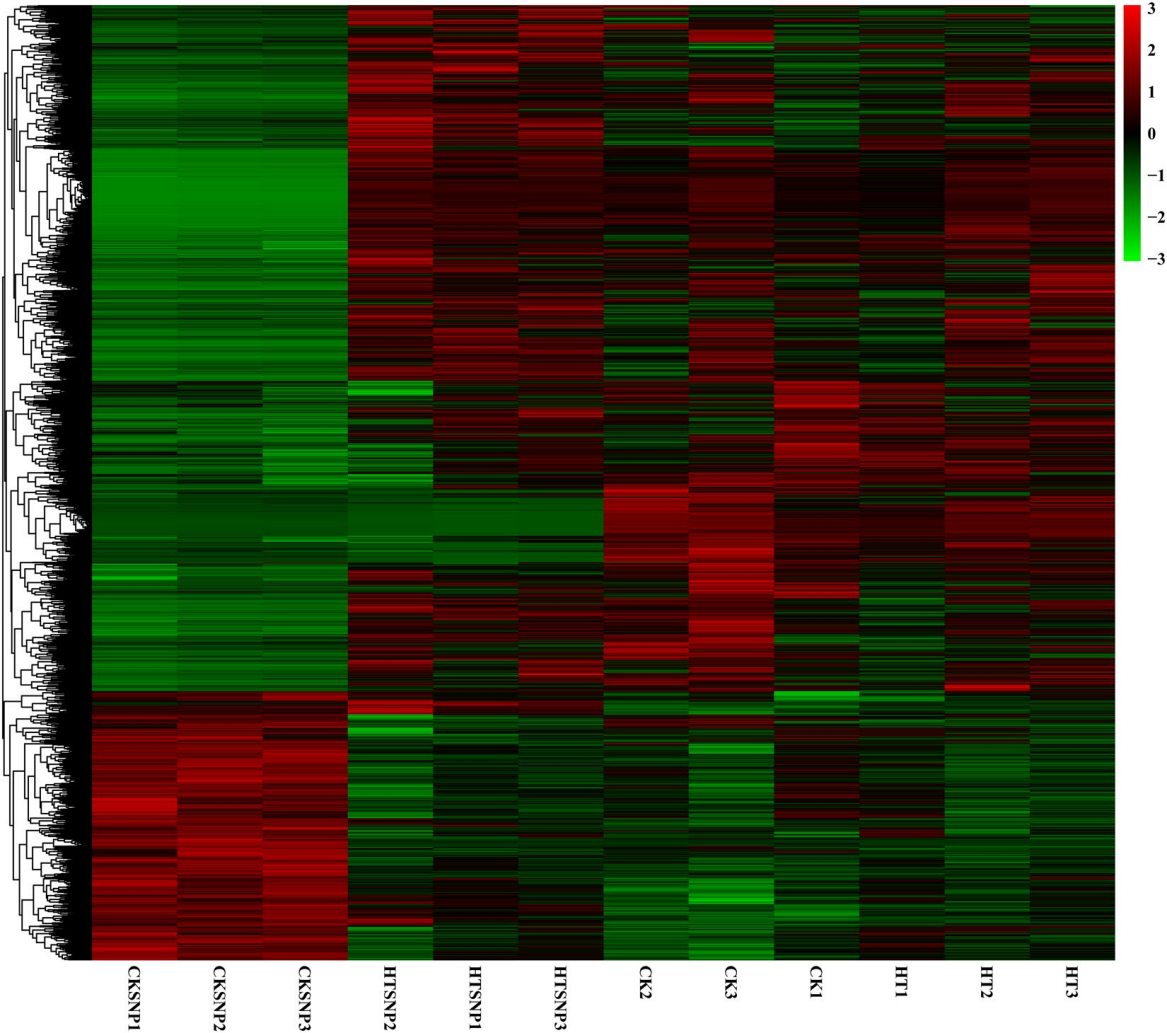
Heatmap and cluster analysis of the DEGs among different samples using complete linkage. The gene expression level increases with color from green to red. CK, *G. oregonense* mycelia treated at 28 °C; HT, *G. oregonense* mycelia treated at 32 °C; HTSNP, *G. oregonense* mycelia treated at 32 °C with 100 µM SNP.

We studied the transcriptional responses of *G. oregonense* to heat stress (32 °C) and the NO-induced gene expression profiles regulating fungal tolerance to abiotic stress. The DEGs among the *G. oregonense* mycelia treated at 28 °C (CK), 32 °C (HT), 28 °C with 100 µM SNP (CKSNP), and 32 °C with 100 µM SNP (HTSNP) were mapped back onto the unigenes respectively (Fig. 6). The expression levels of each unigene were calculated using the RPKM method.

Of the 56,771 unigenes expressed in CK and HT, 484 were significantly differentially expressed in CK and HT (*P* < 0.05), including 197 unigenes up-regulated in HT and 287 in CK. Among the up-regulated genes in HT, HSP genes occupied the largest proportion. A total of eight HSP genes were significantly up-regulated in HT (*P* < 0.05), including two HSP HSS1 genes, two HSP sti1 homologs, two small HSPs, one HSP 16 gene, and one HSP 90 gene (Table 1). Other unigenes, such as “P-loop containing nucleoside triphosphate hydrolase protein gene”, “vacuolar amino acid transporter gene”, “Hsp70 nucleotide exchange factors-like protein gene”, and “probable stress-induced protein gene” were also up-regulated in HT compared with CK (*P* < 0.05). Unigenes related to “FAD/NAD(P)-binding domain-containing protein”, “elongation factor 1-gamma”, and “Tom7-domain-containing protein” were significantly down-regulated in HT (*P* < 0.05) (Table S3).

**Table 1:** Differentially expressed genes coding heat shock proteins (HSPs) between different samples based on RNA-seq analysis.

Gene ID	Gene Length (bp)	HT VS CK		HTSNP VS HT		Gene Description	Accession NO.
		log2 Fold Change	p value	log2 Fold Change	p value		
**DN22896_c0_g1**	758	0.39	8.68E-05			HSP 16	KY781328
**DN25788_c1_g1**	633	0.45	8.64E-03			HSP HSS1	KY781329
**DN25788_c2_g1**	1571	0.42	2.99E-04			HSP HSS1	KY781330
**DN26061_c2_g1**	1174	0.38	4.93E-05			HSP sti1	KY781331
**DN26061_c3_g1**	809	0.38	6.93E-05			HSP sti1	KY781332
**DN26105_c2_g1**	589	0.41	4.82E-03			Small HSP	KY781333
**DN26105_c2_g4**	203	0.43	3.42E-02			Small HSP	KY781334
**DN26764_c0_g1**	5351	0.26	4.86E-08			HSP 90	KY781335
**DN15318_c0_g1**	421			4.19	2.87E-02	HSP 104	KY781336
**DN25759_c0_g1**	2968			2.68	4.89E-05	HSP 104	KY781339
**DN26724_c2_g1**	2881			3.76	3.90E-02	HSP 70	KY781341
**DN26766_c1_g2**	708			2.61	7.55E-04	HSP 78	KY781343
**DN26766_c2_g1**	294			3.29	2.77E-02	HSP 78	781344
**DN26766_c2_g2**	1588			2.68	1.13E-03	HSP 78	KY781345
**DN20233_c0_g1**	733			3.34	1.76E-06	HSP 70	KY781346
**DN24732_c1_g2**	1179			2.84	2.75E-03	HSP 30	KY781347
**DN25210_c3_g3**	482			2.07	5.93E-03	Small HSP C4	KY781348
**DN25520_c0_g2**	545			3.40	1.46E-06	HSP 70	KY781349
**DN25735_c2_g1**	1981			2.04	3.50E-02	HSP 60	KY781350

To assess the functions of the DEGs, functional annotation and GO functional enrichment analysis were adopted for these unigenes. GO functional enrichment analysis showed that only DEGs involved in “oxidoreductase activity” in the category of molecular function were significantly enriched (*P* < 0.05) (Table S6). No DEGs were significantly enriched in the category of cellular components and biological processes, indicating that the two GO terms had no significant effect on the transcriptional response of *G. oregonense* to heat stress.

**Figure 7:**
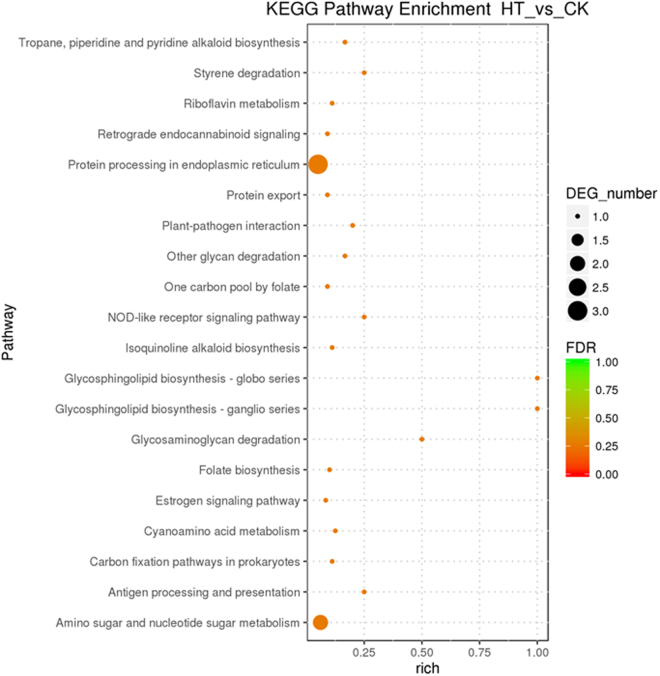
The most significantly enriched KEGG pathways in HT against CK. The y-axis represents different KEGG categories; the x-axis represents the richness factor. The size of the dot indicates the number of DEGs involved in the pathway. The color bars on the right represent the *P*-value of the KEGG pathway.

The regulatory and metabolic pathways relating to the identified unigenes were analyzed using KEGG pathway analysis (Fig. 7). The results showed that pathways involved in “protein processing in endoplasmic reticulum” were significantly enriched in the genetic information processing group (*P* < 0.05) in HT (Table S7). Organismal system pathways related to “NOD-like receptor signaling pathway” and “antigen processing and presentation” were overexpressed in HT (*P* < 0.05). In the category of metabolism, more DEGs were involved in “glycosphingolipid biosynthesis”, “glycosaminoglycan degradation”, “amino sugar and nucleotide sugar metabolism”, and “styrene degradation” in CK (*P* < 0.05). No DEGs were significantly enriched in the category of environmental information processing and cellular processes, indicating that the two KEGG pathways had no significant effect on the transcriptional response of *G. oregonense* to heat stress.

### Transcriptional profiling regulated by NO in response to heat stress

Of the 57,808 unigenes expressed in *G. oregonense* mycelia treated at 32 °C (HT) and mycelia treated at 32 °C with 100 µM SNP (HTSNP), a total of 824 unigenes were significantly differentially expressed between HT and HTSNP (*P* < 0.05), including 375 up-regulated and 449 down-regulated unigenes in *G. oregonense* mycelia treated at 32 °C with 100 µM SNP (HTSNP). In addition to the up-regulated HSP genes in HT compared with CK, many new HSPs were observed the be significantly up-regulated in HTSNP regulated by NO compared with HT (*P* < 0.05), including four unigenes for HSP 78, three HSP 70 genes, two genes for HSP 104, one gene for HSP 30, and one for small HSP C4 (Table 1). Other unigenes related to “NAD(P)-binding protein”, “monooxygenase”, “alcohol dehydrogenase”, “FAD/NAD(P)-binding domain-containing protein”, “Zinc-type alcohol dehydrogenase-like protein”, “putative GTP-binding protein”, and “NAD(P)-binding protein” were also significantly up-regulated in HTSNP compared with HT (Table S4). Other unigenes, including “Glycine-rich RNA-binding protein 7 OS gene”, “Ribulose bisphosphate carboxylase/oxygenase activase”, and “Aquaporin TIP1-2 OS” were significantly down-regulated in HTSNP compared with HT (*P* < 0.05) (Table S4).

GO functional enrichment analysis indicated that many DEGs involved in “misfolded protein binding” and “diacylglycerol O-acyltransferase activity” were significantly enriched (*P* < 0.05) in the category of molecular function (Table S6). In the GO category of biological process, more DEGs were involved in “protein unfolding”, “posttranslational protein targeting to membrane, translocation”, “protein refolding”, and “cellular response to heat”. “Luminal surveillance complex”, “endoplasmic reticulum lumen”, and “nuclear membrane” were more enriched in the category of cellular component.

**Figure 8:**
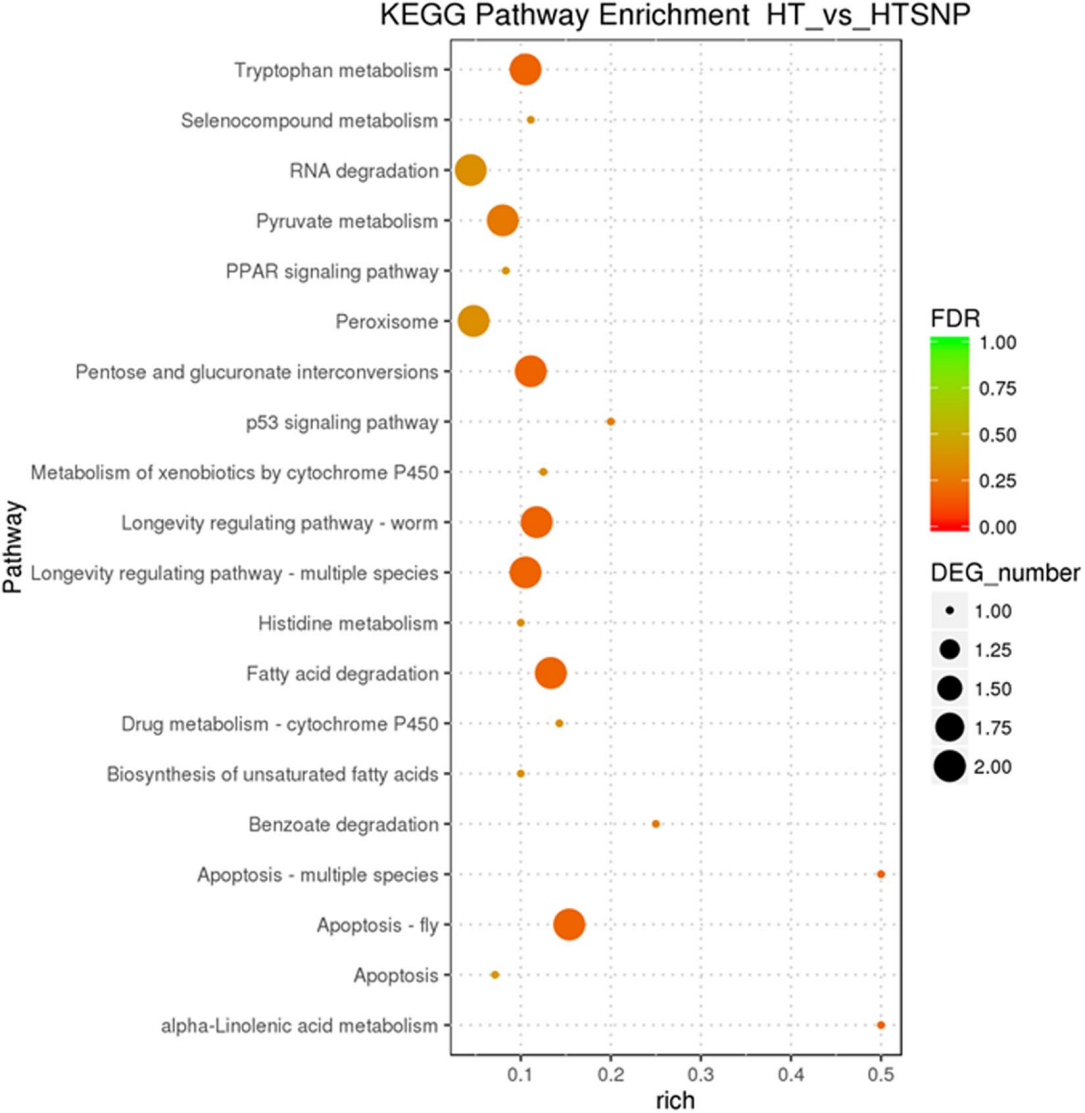
The mostly enriched KEGG pathway in HT against HTSNP. Y-axis represents different KEGG categories; X-axis represents the rich factor. The size of the dot indicates the number of differentially expressed genes (DEGs) involved in the pathway; The color bars on the right represents the P-value of KEGG pathway.

KEGG pathway analysis showed that the pathways involved in “apoptosis – fly” and “apoptosis - multiple species” were significantly enriched in HTSNP in the term of cellular processes (*P* < 0.05) (Fig. 8) (Table S7). Metabolism pathways relating to “fatty acid degradation”, “pentose and glucuronate interconversions”, “alpha-linolenic acid metabolism”, and “tryptophan metabolism” were over-expressed in HTSNP (*P* < 0.05). In the category of organismal systems, more DEGs were involved in “longevity regulating pathway – worm” and “longevity regulating pathway - multiple species” in HTSNP (*P* < 0.05). No DEGs were significantly enriched in the category of environmental information processing and genetic information processing, indicating that these two KEGG pathways had no significant effect on the transcriptional response induced by NO on the regulation of *G. oregonense* tolerance to heat stress.

### Transcriptional profiling regulated by NO under normal conditions

A total of 3,889 unigenes were significantly differentially expressed between CK and CKSNP (*P* < 0.05), including 2,837 unigenes up-regulated and 1,053 down-regulated unigenes in *G. oregonense* mycelia treated at 28 °C with 100 µM SNP (CKSNP). Unigenes related to “NAD-dependent formate dehydrogenase”, “raffinose synthase”, “translation elongation factor”, and “carbohydrate-binding module family 20 protein”, were significantly up-regulated in CKSNP compared with CK (Table S5). Other unigenes, such as “endo-beta-1,4-glucanase”, “aminotransferase”, and “WSC-domain-containing protein” were significantly down-regulated in CKSNP compared with CK (*P* < 0.05) (Table S5).

GO functional enrichment analysis indicated that many DEGs involved in “ribosome”, “structural constituent of ribosome”, “structural molecule activity”, and “intracellular ribonucleoprotein complex” were significantly enriched (*P* < 0.05) in the category of cellular component and molecular function (Table S6). KEGG pathway analysis showed that pathways involved in “Ribosome” and “Plant-pathogen interaction” were significantly enriched in CKSNP in the term of Genetic Information Processing and Organismal Systems (*P* < 0.05) (Table S7). The results showed that in the non-stress environment, NO mainly regulates the structural constituents of ribosomes or structural molecule activities.

### Validation of the DEG results by RT-PCR analysis

To validate gene expression profiles obtained by transcriptome analysis, real-time PCR (RT-PCR) was used to confirm the expression levels of nine selected unigenes, with RPL4 serving as the reference gene (Tables S8 and S9). All of these identified unigenes were successfully amplified and produced a single band. Three unigenes were up-regulated in the CK in comparison to HT. Three exhibited higher expression levels in HTSNP compared to HT. Three were down-regulated in CK compared to HTSNP (Table S8). The genes selected for validation were differentially significantly expressed as calculated by RT-PCR. The expression patterns of these unigenes were consistent with the RNA-Seq analysis, suggesting that the transcriptome analysis was reliable.

## Discussion

*Ganoderma* is an important medicinal fungus with many biological activities, and is also a model macrofungus for studying secondary metabolite synthesis and the abiotic stress response. It has attracted considerable attention in recent years^30,31^. However, the transcriptional responses of *G. oregonense* to heat stress and the transcriptional responses induced by NO resulting in enhanced tolerance to heat stress have not been clarified. Without reference genome, we tried to map the mRNA sequences of this species to the reference genomes of *G. lucidum* in NCBI database. However, the maping rate only reached 61–73%, which indicated that there are great variation in gene content between this strain and *G. lucidum*. The de novo assembled transcriptome of *G. oregonense* SLZ72 revealed that the GC content of *G. oregonense* unigenes was approximately 58.08%, which was higher than in the genomes of *Phellinus linteus* (51.49%)^32^, *Amanita exitialis* (51.58%)^33^, *Lentinula edodes* (48.3%)^34^, *Pleurotus ostreatus* genome (50.9%)^35^, *G. lucidum* G.260125-1 (56.10%)^36^, *G. lucidum* Xiangnong No.1 (55.30%)^37^, and *G. lucidum* BCRC 37177 (55.50%)^38^. However, its GC content was lower than *Auricularia polytricha* (61.3%)^39^. Since there is no reference genome for assembly, the transcripts of *G. oregonense* had to be de novo assembled. The GC content of *G. oregonense* calculated from unigenes of *G. oregonense* should provide a future reference for studying the genetic information of *G. oregonense*, as well as for future studies of its genome and transcriptome. The approximate transcriptome coverage of *G. oregonense* SLZ72 was estimated. The genome sizes of the allied species *G. lucidum* and *G. boninense* ranged from 39.95–60.33 Mb. The 37.8 Gb of the transcriptome sequence data likely represented 627- to 947-fold coverage of the *G. oregonense* SLZ72 genome. Therefore, a large set of *G. oregonense* SLZ72 unigenes can provide adequate information for additional gene investigation.

Increased temperatures associated with global warming have become a major factor affecting the growth of cultivated fungi^40^. *Ganoderma oregonense* is commercially grown in Asia and its yield and quality are reduced by heat stress. It has been reported that the cellular damage induced by HS is mainly due to the excessive accumulation of ROS and lipid peroxidation (LPO) products^41^. As high levels of ROS can result in cytotoxicity, fungi have evolved different strategies for coping with the negative impacts of ROS in response to environmental stress^24,42,43^. The expression of HSPs can play an important role in fungal resistance to HS^40^. So far, the transcriptional response of *G. oregonense* to HS remains unknown, which provides a theoretical basis for elucidating the response of *G. oregonense* to high temperature damage. When *G. oregonense* was subjected to HS (32 °C), its biomass was significantly affected, and 197 unigenes were significantly up-regulated (*P* < 0.05) in the mycelia, including several HSP genes, “P-loop containing nucleoside triphosphate hydrolase protein gene”, “vacuolar amino acid transporter gene”, “nucleotide exchange factors-like protein gene”, and “probable stress-induced protein gene”, indicating that these genes played important roles in HS responses. HSPs are a family of proteins found from bacteria to humans and are produced by cells in response to stressful conditions^44–46^. In *G. oregonense* they were significantly up-regulated when exposed to HS. Zhang *et al*.^40^ found that heat stress modulated the HSP expression of *G. lucidum* via cytosolic Ca^2+^, and this provided additional evidence of the importance of HSPs in the fungal response to HS.

GO functional enrichment analysis and KEGG pathway analysis showed that the DEGs involved in “oxidoreductase activity”, “NOD-like receptor signaling pathway”, “antigen processing and presentation”, and “protein processing in endoplasmic reticulum” were significantly enriched in *G. oregonense* mycelia (*P* < 0.05) subjected to HS, which showed that in *G. oregonense*, enhanced energy metabolism and protein processing occurred in response to adverse conditions. *Streptococcus mutans* also responded to HS by modulating energy metabolism with differentially expressed HSP genes^22^. Other unigenes, such as “vacuolar amino acid transporter gene”, “nucleotide exchange factors-like protein gene”, and “probable stress-induced protein gene” also played important roles in the response of *G. oregonense* to abiotic stress, providing references for the study of the responses of other fungi to abiotic stresses.

When treated with exogenous NO, the biomass of *G. oregonense* mycelia subjected to HS increased. This increase was significant at the concentration of 100 µM SNP, which indicated that NO can enhance the tolerance of *G. oregonense* to HS. This result is consistent with many previous studies^28^. Yu reported that NO can enhance the activity of NOS and NR to reduce oxidative damage and protect the mycelia of *T. harzianum* from HS^47^. The mechanism of NO enhancement of the response of *G. oregonense* to HS has been revealed by RNA-Seq analysis. Unlike non-stress conditions, under which NO mainly regulates the structural constituents of the ribosomes or structural molecule activity, under heat stress a series of genes were activated and some novel HSPs of *G. oregonense* mycelia were significantly up-regulated in HTSNP regulated by NO compared to HT (*P* < 0.05) in order to adapt to the adverse environment. These up-regulated HSPs included HSP 78, HSP 70, HSP 104, HSP 30, and the small HSP C4, indicating that NO activated HSPs to protect *G. oregonense* against abiotic stress. HSPs of various molecular weights were activated together, aiding in protein–protein interactions, protein maintenance, and transportation of proteins across membranes within the cell^48–51^, resulting in a reduction in heat damage. Other unigenes related to “NAD(P)-binding protein”, “monooxygenase”, “alcohol dehydrogenase”, “FAD/NAD(P)-binding domain-containing protein”, “Zinc-type alcohol dehydrogenase-like protein”, “putative GTP-binding protein”, and “NAD(P)-binding protein” were also significantly up-regulated in HTSNP compared with HT. The oxidative damage induced by HS is one of the main factors inhibiting mycelial growth and causing cell death^18,24^. Monooxygenases are enzymes that scavenge ROS in organisms by the concomitant oxidation of NAD(P)H^52^. Alcohol dehydrogenases catalyze the opposite reaction as part of fermentation to ensure a constant supply of NAD+ in bacteria, yeast, and plants^53,54^. The co-expression of these genes could alleviate oxidative damage to *G. oregonense* and increase growth biomass.

GO functional enrichment analysis indicated that the DEGs involved in “misfolded protein binding”, “protein unfolding”, “posttranslational protein targeting to membrane, translocation”, “protein refolding”, and “diacylglycerol O-acyltransferase activity” occupied the largest proportion of the total. The unfolded protein response (UPR), mostly enriched in GO functional analysis, was reported to be a cellular stress response related to the endoplasmic reticulum (ER), which has been found to be conserved in all mammalian species, as well as in yeast and worms^55–57^. Our results showed NO regulated protein processing in organisms responding to abiotic stress. Moreover, “apoptosis – fly”, “apoptosis - multiple species”, “longevity regulating pathway – worm”, and “longevity regulating pathway - multiple species” were found to be over-expressed in HTSNP based on KEGG pathway analysis. Heat-induced, apoptosis-like cell death was also observed in *Pleurotus* species subjected to HS^18^. Many substances are involved in NO-mediated responses to abiotic stress, including trehalose accumulation and reactive oxygen scavenging enzymes^28,47,58^. This is the first study of the gene expression of *G. oregonense* simultaneously exposed to NO and abiotic stress. Our results can be applied to similar studies on other fungal studies. Further research is needed to reveal the mechanism by which NO allows other fungi to tolerate abiotic stresses and their transcriptional regulation mechanisms. This will provide a basis for the improved cultivation and molecular breeding of macrofungi.

## Materials and Methods

### Sample collection and preparation

*G. oregonense* SLZ72 was introduced by an expert from the United States through cooperation and successfully cultivated in southwestern China. *G. oregonense* mycelia were obtained from cultivated strain and identified by the authors based on morphology (the cream to pinkish-buff cap, absence of concentric growth zones and melanoid bands in mature fruiting body, the white pore color at maturity, the ellipsoid or coarsely echinulate basidiospore) and molecular analysis (GenBank accession numbers KY781351 and KY781352). The species is now preserved in the collection center of Institute of plant protection, Sichuan Academy of Agricultural Sciences (specimen: SLZ72). Two rounds of experiments were performed. The first aimed to assess the effect of heat stress on growth rate in order to evaluate the temperature at which the species began to show evidence of heat stress. From these preliminary tests, mycelia were selected for the “heat treated” group for further testing the transcriptional response of *G. oregonense* to heat stress, as well as assessing the mechanism by which NO enhances heat stress tolerance of fungi at the gene expression level. The obtained mycelia were first incubated in potato dextrose agar medium (PDA) at 28 °C for 6 d, and then 10 pieces (a 5 mm diameter punch) of the mycelia from the solid medium were transferred to 200 mL liquid potato dextrose medium (LPD) in a 500 mL flask. The cultures were incubated with shaking at 110 rmp and 24 °C, 26 °C, 28 °C, 30 °C, 32 °C, and 34 °C for 6 d respectively, and fresh mycelial biomass was tested. In the preliminary heat treatment experiments, mycelia were subjected to heat stress at 32 °C and different concentrations (0, 50, 100, 200, 300 µM) of SNP (sodium nitroprusside dihydrate, C_5_H_4_FeN_6_Na_2_O_3_) were then added to heat-stressed mycelia, which were incubated at 110 rpm and harvested after 6 d for fresh weight (FW) determination. Potassium ferrocyanide (K_4_[Fe(CN)_6_]), an analog of SNP which does not release NO, was applied as a negative control of SNP^29^. SNP and K_4_[Fe(CN)_6_] were directly added into the flasks after filter sterilization. Each treatment was replicated in triplicate under the same experimental conditions. The mycelia were harvested after 6 d for RNA extraction and transcriptome sequencing.

### RNA isolation, library preparation, and sequencing

RNA was prepared using the Qiagen RNeasy Mini Kit (Qiagen, Germany) with DNAase treatment according to manufacturer’s instructions. The quality and quantity of extracted RNA were assessed using 1% agarose gels and an Agilent 2100 Bioanalyzer (Agilent Technologies, CA, USA). RNA with a RIN value greater than 9.0 was used for transcriptome library production. RNA sequencing libraries were generated using the TruSeq RNA Sample Preparation Kit (Illumina, San Diego, CA, USA) according to instructions. Briefly, the mRNA was purified from total RNA using poly-T oligo-attached magnetic beads and short fragments were obtained under elevated temperature by adding an Illumina proprietary fragmentation buffer. First strand cDNA was synthesized using random hexamer primer and M-MuLV Reverse Transcriptase (RNase H-). Second strand cDNA synthesis was performed using DNA Polymerase I and RNase H. Remaining overhangs were converted into blunt ends via exonuclease/polymerase^59^. To select cDNA fragments of the preferred 200~300 bp length, the library fragments were purified with the AMPure XP system (Beckman Coulter, Beverly, USA). After adenylation of the 3’ ends of the DNA fragments, NEBNext adaptors, with hairpin loop structures, were ligated to prepare them for hybridization. Then 3 μL USER Enzyme (NEB, USA) was used with size-selected, adaptor-ligated cDNA at 37 °C for 15 min followed by 5 min at 95 °C prior to PCR. PCR was performed with Phusion High-Fidelity DNA polymerase, Universal PCR primers, and Index (X) Primer. PCR products were purified (AMPure XP system) and library quality was assessed on an Agilent Bioanalyzer 2100 system. The library was sequenced with the Illumina HiSeq™ 2000. The entire process was commissioned and completed by the Shanghai Personal Biotechnology Co.

### Transcriptome assembly and gene functional annotation

Raw data (raw reads) of fastq format were first processed through in-house perl scripts. In this step, clean data (clean reads) were obtained by removing reads containing adapters, reads containing poly-Ns, and low quality reads from the raw data. At the same time, Q20, Q30, GC-content, and the sequence duplication level of the clean data were calculated. All downstream analyses were based on clean data with high quality. The left files (read1 files) from all libraries/samples were pooled into one large left.fq file, and right files (read2 files) into one large right.fq file. Transcriptome assembly was accomplished based on the left.fq and right.fq using Trinity with min_kmer_cov set to 2 by default and all other parameters set to default^60^. Gene functions were annotated based on the following databases: Nr (NCBI non-redundant protein sequences); eggNOG (Non-supervised Orthologous Groups); Swiss-Prot (A manually annotated and reviewed protein sequence database); GO (Gene Ontology) and KEGG (the Kyoto Encyclopedia of Genes and Genomes).

### Differential expression analysis

Gene expression levels for each sample were estimated by RSEM and clean data were mapped back onto the assembled transcriptome^61^. Read counts for each gene were obtained from the mapping results. Differential expression analysis of two conditions/groups was performed using the DESeq R package (1.10.1). DESeq provides statistical routines for determining differential expression in digital gene expression data using a model based on the negative binomial distribution. The resulting *P*-values were adjusted using the Benjamini and Hochberg approach for controlling the false discovery rate. Genes with an adjusted *P*-values < 0.05 found by DESeq were designated as differentially expressed.

### GO and KEGG enrichment analysis

GO enrichment analysis of the DEGs was accomplished using the GOseq R package-based Wallenius non-central hyper-geometric distribution, which adjusts for gene length bias in DEGs^62^. KEGG is a database resource for elucidating high-level functions and utilities of the biological system^63^, such as the cell, the organism, and the ecosystem. KEGG is used with molecular-level information, especially large-scale molecular datasets generated by genome sequencing and other high-throughput experimental technologies (http://www.genome.jp/kegg/). We used KOBAS software to test the statistical enrichment of differential expression genes in KEGG pathways^64^.

### Experimental validation of gene expression using RT-qPCR

The expression of nine unigenes identified by RNA-Seq was validated using real time PCR (RT-qPCR) analysis. Briefly, about 3 mg of total RNA of each sample was reverse-transcribed using Super RT Kit (TaKaRa) according to manufacturer’s instructions. The gene-specific primers were designed based on the unigene sequences. RPL4 primers were used as endogenous loading controls for testing the validity of template preparation^65^. The expression of each gene was confirmed by at least three rounds of independent RT-PCR reactions. Relative quantification of target gene expression was evaluated using the comparative cycle threshold (CT) method as previously described by Livak & Schmittgen^66^. The ΔCT value was determined by subtracting the target CT of each sample from its respective RPL4 CT value. Differences in expression of the target genes were determined by 2^−ΔΔCT^. Data were expressed as arithmetic means ± SD. Comparisons between different samples were performed using Student’s *t*-tests and a *P*-value less than 0.05 was considered as statistically significant.

### Statistical analysis

Data of this study are presented as means ± SD of three replicates for each treatment. Statistical analysis was carried out by one-way analysis of variance (ANOVA) using SPSS 19.0. Least significant difference (LSD) was performed to test if the ANOVA result between different treated groups was significant at *P* < 0.05.

### Data availability statement

All the raw sequence data is stored in The Sequence Read Archive (SRA) database (accession number: SRR5274645- SRR5274653).

## Electronic supplementary material

Supplementary figures 1-2 and tables 1–9
