# Supplementary material for: Transcriptional profiling provides new insights into the role of nitric oxide in enhancing Ganoderma oregonense resistance to heat stress
Source: Sci Rep. 2017 Nov 16;7:15694. doi: 10.1038/s41598-017-15340-6 (PMC5691203; doi:10.1038/s41598-017-15340-6)
Supplement: Supplementary file 1 — Supplementary figures 1-2 and tables 1–9 [file 41598_2017_15340_MOESM1_ESM.pdf]

Transcriptional profiling provides new insights into the role of nitric oxide in enhancing *Ganoderma oregonense* resistance to heat stress

Running title: NO enhances *G. oregonense* tolerance to heat stress

Cheng Chen[1], Qiang Li[+2,3], Qiangfeng Wang[2], Daihua Lu[1], Hong Zhang[1]*, Jian Wang[1], Rongtao Fu[1]

[1] *Institute of plant protection, Sichuan Academy of Agricultural Sciences, Chengdu 610066, P.R. China*

[2] *Biotechnology and Nuclear Technology Research Institute, Sichuan Academy of Agricultural Sciences, Chengdu 610061, Sichuan, P.R. China*

[3] *College of Life Science, Sichuan University, Chengdu 610065, Sichuan, P.R. China*

*Corresponding author: Hong Zhang

E-mail: 1622680749@qq.com; Phone: 86-028-84592187;

Present address: Sichuan Academy of Agricultural Sciences,

20 # Jingjusi Rd, Chengdu 610066, Sichuan, China.

[+] Cheng Chen and Qiang Li contributed equally to this work

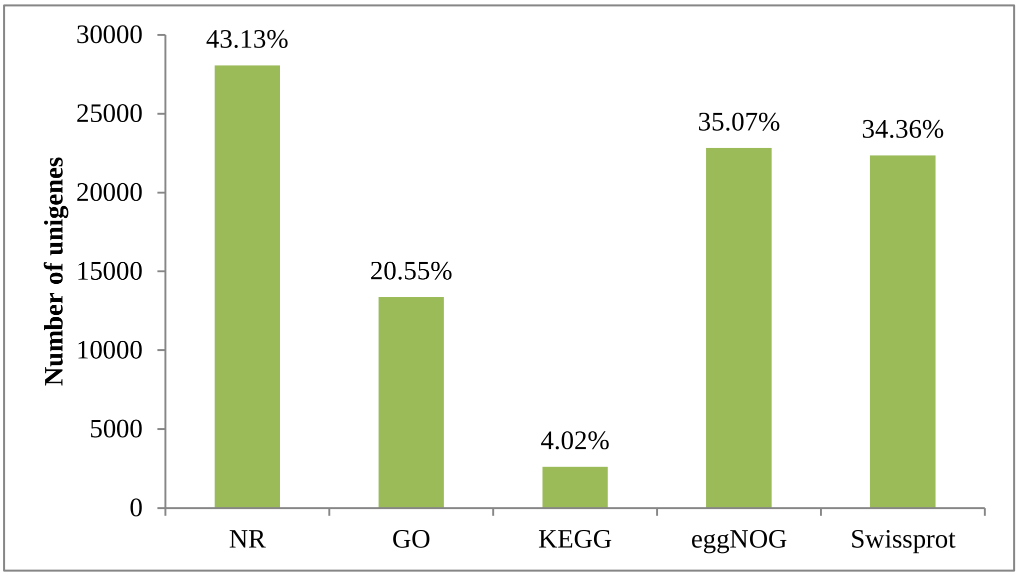

Figure S1 Number of unigenes annotated against five databases.

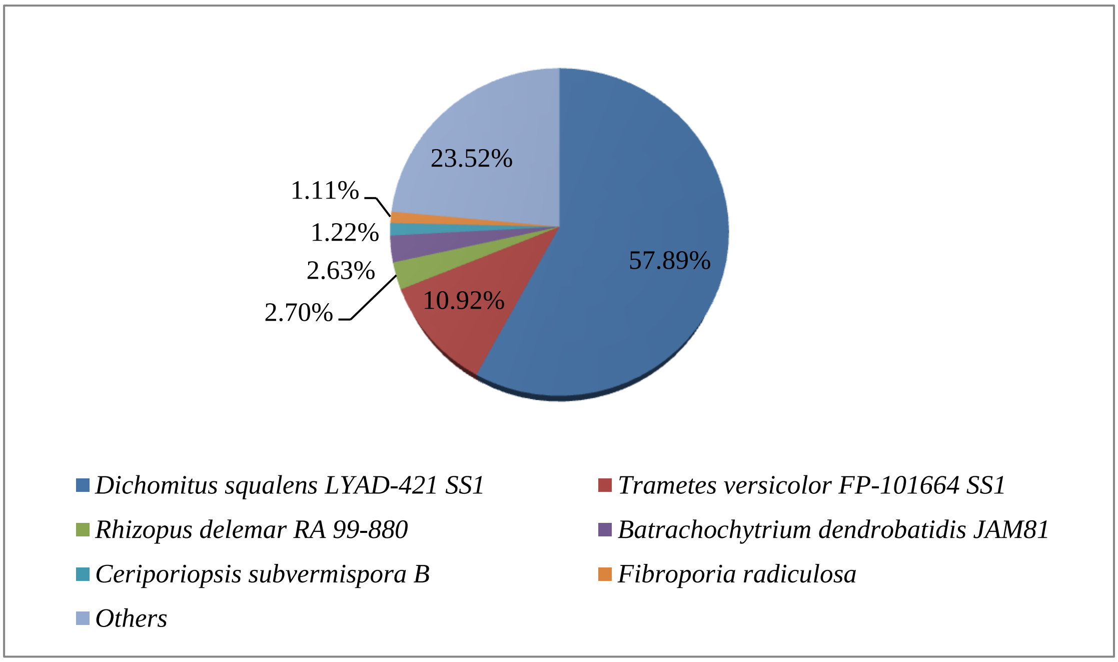

Figure S2 Species distribution in Basidiomycota of transcriptome sequences with top BLASTx hits against nr database.

Table S1 Throughput and quality of Illumina sequencing of *Ganoderma oregonense* transcriptome.

Sample	Raw Reads	Clean Reads	Clean Bases	Error (%)	Q20 (%)	Q30 (%)	GC (%)
CK1	2.79*107	2.76*107	4.00*109	1.44*10-3	98.2	95.14	57.56
CK2	2.96*107	2.92*107	4.32*109	1.29*10-3	97.95	94.57	55.03
CK3	2.94*107	2.89*107	4.21*109	1.43*10-3	98.07	94.82	56.25
CKSNP1	3.92*107	3.88*107	5.72*109	2.79*10-3	97.94	94.6	58.87
CKSNP2	3.74*107	3.70*107	5.47*109	3.03*10-3	97.77	94.18	59.00
CKSNP3	3.67*107	3.63*107	5.37*109	2.49*10-3	97.5	93.63	59.06
HT1	2.83*107	2.80*107	4.10*109	1.33*10-3	98.05	94.75	59.19
HT2	3.13*107	3.09*107	4.52*109	1.28*10-3	97.94	94.5	56.52
HT3	2.81*107	2.78*107	4.07*109	1.48*10-3	98.21	95.12	56.97
HTSNP1	2.99*107	2.96*107	4.37*109	1.49*10-3	98.06	94.77	58.85
HTSNP2	2.79*107	2.76*107	4.04*109	1.43*10-3	98.02	94.69	59.35
HTSNP3	2.90*107	2.88*107	4.21*109	1.42*10-3	98.16	95.01	59.61

CK, *G. oregonense* mycelia treated at 28 ℃; CKSNP, *G. oregonense* mycelia treated at 28 ℃ with 100 μM SNP; HT, *G. oregonense* mycelia treated at 32 ℃; HTSNP, *G. oregonense* mycelia treated at 32 ℃ with 100 μM SNP

Table S2 Length distribution of the contigs, transcripts and unigenes of *Ganoderma oregonense*

	**Contig**	**Transcript**	**Unigene**
Total Length (bp)	6.49*107	1.23*108	4.49*107
Max. Length (bp)	15,441	15,439	15,439
Mean Length (bp)	348	1107	767
N50 (bp)	626	1895	1512
N90 (bp)	136	441	286

Table S3 Differentially expressed genes (DEGs) between HT and CK (pvalue ≤ 0.0001).

ID	foldchange	pvalue	Length	NR Description
**UP-regulated in *Ganoderma oregonense* mycelium subjected to heat stress (HT)**				
DN39269_c0_g1	0.19	3.02*10-8	392	-
DN17391_c0_g1	0.15	4.19*10-8	800	P-loop containing nucleoside triphosphate hydrolase protein
DN20661_c1_g1	0.29	2.34*10-7	2214	Vacuolar amino acid transporter 1 OS
DN23784_c2_g1	0.31	1.18*10-6	977	Hsp70 nucleotide exchange factor FES1 OS
DN23012_c1_g2	0.23	1.73*10-6	1613	Basic form of pathogenesis-related protein 1 OS
DN23784_c1_g1	0.34	2.76*10-6	652	predicted protein [Fibroporia radiculosa]
DN10696_c0_g1	0.29	1.02*10-5	656	hypothetical protein DICSQDRAFT_124759
DN26608_c2_g2	0.3	2.43*10-5	415	-
DN17910_c0_g1	0.28	2.95*10-5	1318	Transaldolase OS
DN26266_c3_g1	0.16	4.03*10-5	790	-
DN26608_c4_g1	0.29	5.20*10-5	694	hypothetical protein PHACADRAFT_101076
DN21579_c0_g1	0.33	6.03*10-5	1494	LIP_RHIOR Lipase OS
**Down-regulated in *Ganoderma oregonense* mycelium subjected to heat stress (HT)**				
DN19739_c0_g3	10.15	5.74*10-15	397	-
DN26323_c1_g4	5.14	8.06*10-11	944	-
DN26299_c2_g4	11.03	2.92*10-10	345	FAD/NAD(P)-binding domain-containing protein
DN26323_c0_g1	4.21	1.65*10-8	605	-
DN25698_c1_g6	3.29	3.63*10-6	297	-
DN19777_c0_g3	20.31	3.88*10-6	703	-
DN16857_c0_g2	4.56	5.39*10-6	1063	hypothetical protein DICSQDRAFT_157787
DN8253_c0_g1	5.98	1.15*10-5	667	hypothetical protein PHACADRAFT_169427
DN19739_c0_g2	4.37	2.93*10-5	393	-
DN18126_c1_g2	3.26	4.86*10-5	279	Tom7-domain-containing protein

DN25698_c1_g12	2.77	7.22*10-5	760	hypothetical protein DICSQDRAFT_138718
DN8253_c0_g2	4.88	7.70*10-5	612	hypothetical protein PHACADRAFT_169427
DN18365_c0_g1	4.72	8.01*10-5	466	elongation factor 1-gamma
DN25698_c1_g14	3.06	8.84*10-5	362	hypothetical protein DICSQDRAFT_138718
DN7554_c0_g2	9.38	9.8*10-5	356	-

Differentially expressed heat shock proteins (HSPs) are not included in this table.

Table S4 Differentially expressed genes (DEGs) between HTSNP and HT (pvalue ≤ 0.0001).

ID	foldchange	pvalue	Length	NR Description
**UP-regulated in *Ganoderma oregonense* mycelium treated at 32 ℃ with 100 μM SNP (HTSNP)**				
DN22553_c0_g1	7.67	2.81*10-12	1495	NAD(P)-binding protein
DN26105_c1_g1	8.14	2.59*10-9	910	monooxygenase [Dichomitus squalens LYAD-421 SS1]
DN18358_c0_g1	5.95	1.27*10-8	1376	hypothetical protein PHACADRAFT_145396
DN25853_c0_g5	346.95	2.66*10-8	344	alcohol dehydrogenase [Dichomitus squalens LYAD-421 SS1]
DN4193_c0_g1	5.65	1.09*10-7	1094	FAD/NAD(P)-binding domain-containing protein
DN25853_c0_g2	212.23	2.61*10-7	1008	Zinc-type alcohol dehydrogenase-like protein
DN25853_c0_g1	188.85	6.77*10-7	947	2-alkenal reductase (NADP(+)-dependent) OS
DN25520_c0_g5	3.46	9.61*10-7	970	glucose-regulated protein homolog OS
DN18321_c0_g1	3.46	1.05*10-6	1481	hypothetical protein PHACADRAFT_252264
DN33274_c0_g1	9.77	1.42*10-6	342	-
DN21402_c0_g1	28.08	1.99*10-6	1328	Metallo-hydrolase/oxidoreductase
DN23973_c0_g3	4.88	4.3*10-6	625	-
DN25520_c0_g11	3.22	4.48*10-6	393	putative GTP-binding protein
DN25520_c0_g8	3.16	8.01*10-6	393	putative GTP-binding protein
DN22628_c0_g1	101.75	2.66*10-5	1487	NAD-P-binding protein [Trametes versicolor FP-101664 SS1]
DN23483_c0_g1	8.3	2.92*10-5	817	NAD-P-binding protein [Trametes versicolor FP-101664 SS1]
DN24006_c0_g1	3.43	3.12*10-5	2852	hypothetical protein SCHCODRAFT_81041
DN21758_c0_g2	5.11	3.88*10-5	763	-
DN25735_c2_g2	5.68	4.82*10-5	384	-
DN26105_c1_g2	3.66	4.87*10-5	690	monooxygenase FAD-binding protein
DN25759_c0_g2	3.22	6.49*10-5	275	-
DN19769_c0_g1	46.15	9.68*10-5	315	hypothetical protein DICSQDRAFT_157052
**Down-regulated in *Ganoderma oregonense* mycelium treated at 32 ℃ with 100 μM SNP (HTSNP)**				

DN12775_c0_g1	0	8.11*10-13	417	Stress-induced protein KIN2 OS
DN20055_c1_g1	0	1.4*10-11	366	Ribulose bisphosphate carboxylase small chain 3B
DN39421_c0_g1	0	1.56*10-9	1160	Hygromycin-B 4-O-kinase OS
DN14121_c0_g3	0	1.78*10-9	608	Glycine-rich RNA-binding protein 7 OS
DN11848_c0_g1	0	2.48*10-9	722	Ribulose bisphosphate carboxylase/oxygenase activase
DN13185_c1_g1	0	3.71*10-9	560	Probable fructose-bisphosphate aldolase 2
DN14121_c0_g4	0	3.89*10-9	693	Glycine-rich RNA-binding protein 7 OS
DN556_c0_g1	0	9.72*10-9	290	hypothetical protein SERLA73DRAFT_80747
DN30295_c0_g1	0	1.32*10-8	586	Aquaporin TIP1-2 OS
DN28531_c0_g1	0	1.85*10-8	436	-
DN5812_c0_g2	0	2.38*10-8	522	proline-rich protein OS
DN15400_c0_g3	0.01	3.41*10-8	371	hypothetical protein MGL_3949
DN13990_c0_g2	0	5.11*10-8	635	-
DN24547_c3_g2	0.03	9.39*10-8	2872	Uncharacterized protein ORF91 OS
DN28020_c0_g1	0	1*10-7	484	-
DN28705_c0_g1	0	2.84*10-7	608	Aminomethyltransferase, mitochondrial OS
DN15515_c0_g1	0.02	3.01*10-7	327	Putative uncharacterized protein ART2 OS
DN40443_c0_g1	0	3.12*10-7	479	Metallothionein-like protein 2A OS
DN2827_c0_g1	0	3.32*10-7	322	-
DN31096_c0_g1	0	9.66*10-7	457	-
DN26344_c2_g13	0.07	1.46*10-6	927	-
DN31430_c0_g1	0	2.24*10-6	295	-
DN7209_c0_g2	0	2.64*10-6	351	Metallothionein-like protein type 3 OS
DN30334_c0_g1	0	2.82*10-6	754	Aquaporin TIP2-1 OS
DN2200_c0_g1	0	2.91*10-6	544	40S ribosomal protein Sa-1 OS
DN2683_c0_g1	0	3.24*10-6	429	Aquaporin TIP1-1 OS

DN11615_c0_g1	0	4.84*10-6	383	Non-specific lipid-transfer protein 5 OS
DN9598_c0_g2	0	5.31*10-6	348	-
DN3428_c0_g1	0	5.71*10-6	404	60S ribosomal protein L29-1 OS
DN10158_c0_g1	0	7.87*10-6	891	Fructose-bisphosphate aldolase, cytoplasmic isozyme OS
DN24547_c3_g3	0.04	7.93*10-6	296	hypothetical protein SERLA73DRAFT_67483
DN16950_c0_g1	0	8.37*10-6	291	hypothetical protein CERSUDRAFT_61093
DN888_c0_g1	0.01	1.12*10-5	300	hypothetical protein MYCGRDRAFT_74248
DN17600_c4_g1	0	1.18*10-5	487	-
DN16450_c0_g1	0	1.57*10-5	777	-
DN16599_c0_g2	0.05	1.67*10-5	428	hypothetical protein AGABI1DRAFT_49393
DN13990_c0_g1	0	1.93*10-5	635	-
DN5832_c0_g1	0	2.61*10-5	406	Non-specific lipid-transfer protein 1 OS
DN14407_c0_g2	0	3.73*10-5	390	-
DN33351_c0_g1	0	3.74*10-5	595	-
DN530_c0_g1	0	3.96*10-5	455	lipid transfer protein [Uromyces hobsonii]
DN39376_c0_g1	0	4.07*10-5	428	Triosephosphate isomerase
DN38587_c0_g1	0	4.37*10-5	332	Metallothionein-like protein 2B OS
DN6434_c0_g1	0	4.63*10-5	808	Polyadenylate-binding protein 2 OS
DN14407_c0_g1	0	4.67*10-5	402	-
DN43291_c0_g1	0	6*10-5	687	40S ribosomal protein S8-1 OS
DN4870_c0_g1	0	6.03*10-5	444	CBS domain-containing protein YhcV OS
DN3851_c0_g1	0	6.29*10-5	539	Glutamine synthetase, chloroplastic/mitochondrial OS
DN2022_c0_g1	0	6.69*10-5	390	-
DN9388_c0_g1	0	6.72*10-5	724	-
DN8406_c0_g1	0	6.75*10-5	363	-
DN13492_c0_g2	0.1	7.78*10-5	264	hypothetical protein NEUTE2DRAFT_76219

DN20004_c0_g1	0.3	8.29*10-5	1584	hypothetical protein CERSUDRAFT_118876
DN14121_c0_g5	0	9.15*10-5	594	hypothetical protein BATDEDRAFT_27077

Differentially expressed heat shock proteins (HSPs) are not included in this table.

Table S5 Differentially expressed genes (DEGs) between CKSNP and CK (pvalue ≤ 1E-9).

ID	foldchange	pvalue	Length	NR Description
**UP-regulated in *Ganoderma oregonense* mycelium treated at 28 ℃ with 100 μM SNP (CKSNP)**				
c18728_g1	30.96470946	3.73856E-21	1136	NAD-dependent formate dehydrogenase [Dichomitus squalens LYAD-421 SS1]
c36706_g1	Inf	2.29689E-20	1516	-
c24097_g1	Inf	2.31842E-18	5239	-
c25475_g1	Inf	1.23572E-17	6850	-
c23243_g1	Inf	4.50623E-17	1047	-
c14551_g2	Inf	3.93651E-16	1572	-
c14810_g1	Inf	1.42693E-15	561	-
c17166_g1	Inf	3.75933E-15	1600	-
c13741_g1	Inf	9.12049E-15	562	-
c21067_g1	Inf	1.32247E-14	913	-
c53613_g1	Inf	1.91199E-14	1380	-
c18225_g2	Inf	3.22542E-14	1816	hypothetical protein CIRG_10393 [Coccidioides immitis RMSCC 2394]
c13624_g1	Inf	3.70159E-14	710	-
c17474_g1	Inf	4.05198E-14	1075	class iv chitinase [Nosema apis BRL 01]
c9767_g1	Inf	5.13399E-14	1557	hypothetical protein SPPG_00617 [Spizellomyces punctatus DAOM BR117]
c12976_g1	Inf	1.30671E-13	661	-
c11498_g1	Inf	1.33942E-13	1101	-
c20279_g1	Inf	1.51259E-13	733	-
c9093_g1	Inf	2.02929E-13	574	-
c10031_g1	Inf	2.40081E-13	473	-
c10353_g1	Inf	2.56338E-13	2761	raffinose synthase [Coccidioides immitis H538.4]
c13632_g1	Inf	4.82428E-13	1271	hypothetical protein SPPG_01926 [Spizellomyces punctatus DAOM BR117]
c22119_g1	Inf	5.69734E-13	6287	-

c8549_g1	Inf	6.65667E-13	844	YALI0E34111p [Yarrowia lipolytica]
c12719_g1	Inf	7.40077E-13	675	-
c16051_g1	Inf	7.49138E-13	633	-
c16682_g3	Inf	9.62044E-13	856	-
c19154_g1	Inf	1.02021E-12	710	-
c11425_g1	Inf	1.42406E-12	1013	-
c12214_g1	Inf	1.46839E-12	557	-
c15765_g1	Inf	1.73501E-12	2002	hypothetical protein GLOINDRAFT_199136 [Rhizophagus irregularis DAOM 181602]
c26754_g1	Inf	1.82386E-12	683	-
c15000_g1	Inf	2.49724E-12	417	hypothetical protein RHOBADRAFT_19446 [Rhodotorula graminis WP1]
c15745_g1	Inf	3.02685E-12	812	-
c12279_g1	Inf	3.24999E-12	555	-
c13580_g2	Inf	3.44078E-12	723	hypothetical protein PGTG_14957 [Puccinia graminis f. sp. tritici CRL 75-36-700-3]
c959_g1	Inf	4.20905E-12	718	-
c10247_g1	Inf	4.33826E-12	521	-
c25504_g2	14.08766574	4.70773E-12	843	hypothetical protein DICSQDRAFT_170772 [Dichomitus squalens LYAD-421 SS1]
c16569_g1	Inf	5.38084E-12	1436	hypothetical protein SPPG_01813 [Spizellomyces punctatus DAOM BR117]
c23067_g1	Inf	5.57373E-12	1857	-
c42152_g1	Inf	6.79426E-12	1164	hygromycin-B [Coccidioides posadasii C735 delta SOWgp]
c16879_g1	Inf	7.79678E-12	1486	YALI0D14300p [Yarrowia lipolytica]
c13760_g1	Inf	8.34097E-12	1525	-
c16109_g1	Inf	1.06129E-11	605	-
c14793_g1	Inf	1.10413E-11	930	calmodulin [Spizellomyces punctatus DAOM BR117]
c49119_g1	Inf	1.10438E-11	339	-
c12558_g1	Inf	1.21398E-11	847	-
c30904_g1	Inf	1.2271E-11	857	-

c9032_g1	Inf	1.30098E-11	437	-
c9655_g2	Inf	1.5802E-11	602	-
c11599_g1	Inf	1.72919E-11	1053	ras protein [Lichtheimia corymbifera JMRC:FSU:9682]
c16484_g1	Inf	1.73409E-11	1788	aspartic-type endopeptidase [Blastomyces gilchristii SLH14081]
c9858_g1	Inf	2.18262E-11	472	-
c16072_g1	Inf	2.36595E-11	1427	thioredoxin reductase [Sistotremastrum niveocremeum HHB9708]
c11866_g1	Inf	2.6356E-11	1534	hypothetical protein BATDEDRAFT_15114 [Batrachochytrium dendrobatidis JAM81]
c3338_g1	Inf	3.20379E-11	624	-
c13495_g1	Inf	3.40377E-11	1070	Ras-like protein [Phialophora attae]
c53740_g1	Inf	3.60836E-11	392	-
c24761_g2	Inf	3.91952E-11	2142	-
c21048_g1	16.30415147	4.41171E-11	1552	hypothetical protein DICSQDRAFT_175651 [Dichomitus squalens LYAD-421 SS1]
c3727_g1	Inf	4.71516E-11	537	-
c4735_g1	Inf	4.78648E-11	455	-
c25579_g2	Inf	5.89665E-11	1338	hypothetical protein BATDEDRAFT_36305 [Batrachochytrium dendrobatidis JAM81]
c11191_g1	Inf	5.90698E-11	426	-
c22793_g1	7.295714691	6.00138E-11	2511	hypothetical protein DICSQDRAFT_69652 [Dichomitus squalens LYAD-421 SS1]
c19804_g1	Inf	6.43354E-11	1431	-
c9650_g1	Inf	6.50888E-11	531	-
c14832_g1	Inf	7.33118E-11	531	-
c16003_g1	Inf	9.28867E-11	2044	carbohydrate-binding module family 20 protein [Baudoinia panamericana UAMH 10762]
c9768_g1	Inf	1.05344E-10	535	-
c15398_g1	913.6813902	1.34231E-10	442	hypothetical protein TSTA_040370 [Talaromyces stipitatus ATCC 10500]
c16341_g1	Inf	1.39752E-10	6190	hypothetical protein SPPG_07224 [Spizellomyces punctatus DAOM BR117]
c12381_g1	Inf	1.6357E-10	460	-
c15131_g1	Inf	1.75751E-10	1542	hypothetical protein AMAG_13027 [Allomyces macrogynus ATCC 38327]

c19093_g1	Inf	1.79422E-10	742	-
c8425_g1	Inf	2.11378E-10	573	-
c14551_g1	Inf	2.19302E-10	1544	-
c16751_g1	Inf	2.44241E-10	2045	-
c9541_g1	Inf	2.50915E-10	595	-
c11943_g1	Inf	2.67355E-10	666	hypothetical protein CGLO_10530 [Colletotrichum gloeosporioides Cg-14]
c10623_g1	Inf	3.0749E-10	769	-
c1764_g1	Inf	3.10411E-10	347	-
c18298_g1	Inf	3.12559E-10	1010	-
c17208_g1	864.0349336	3.29423E-10	1069	hydrolase, partial [Mucor ambiguus]
c14723_g1	Inf	3.37573E-10	965	-
c12142_g1	Inf	3.55965E-10	482	-
c19454_g1	Inf	3.70162E-10	1021	-
c13699_g1	Inf	3.77963E-10	508	-
c14810_g2	Inf	4.01151E-10	569	-
c9728_g1	Inf	4.43774E-10	556	-
c7443_g1	Inf	4.6817E-10	603	-
c18221_g1	Inf	4.92085E-10	990	-
c13310_g1	Inf	5.72994E-10	1001	hypothetical protein VC83_06664 [Pseudogymnoascus destructans]
c13195_g1	Inf	5.93376E-10	852	60S ribosomal protein L10 [Auricularia subglabra TFB-10046 SS5]
c14305_g1	Inf	6.78327E-10	1478	-
c19416_g1	235.7991948	6.83855E-10	646	hypothetical protein M408DRAFT_317305, partial [Serendipita vermifera MAFF 305830]
c14006_g1	Inf	7.62657E-10	1350	translation elongation factor [Mortierella elongata AG-77]
c12481_g1	Inf	8.09964E-10	664	-
c12512_g1	Inf	8.39743E-10	781	-
c13002_g1	Inf	9.76162E-10	348	-

**Down-regulated in *Ganoderma oregonense* mycelium treated at 28 ℃ with 100 μM SNP (CKSNP)**				
c24713_g2	0.037581233	5.26268E-22	2921	hypothetical protein DICSQDRAFT_135520 [Dichomitus squalens LYAD-421 SS1]
c25538_g1	0.059964615	2.39352E-20	5652	hypothetical protein BN946_scf184785.g53 [Trametes cinnabarina]
c20758_g1	0.070489732	1.25113E-18	1825	endo-beta-1,4-glucanase [Dichomitus squalens LYAD-421 SS1]
c20956_g1	0.072541673	2.12885E-18	2703	hypothetical protein DICSQDRAFT_158402 [Dichomitus squalens LYAD-421 SS1]
c22256_g1	0.075590702	2.21448E-18	1803	nucleotide exchange factors-like protein [Dichomitus squalens LYAD-421 SS1]
c18622_g1	0.048309955	2.2209E-18	1918	WSC-domain-containing protein [Dichomitus squalens LYAD-421 SS1]
c23725_g1	0.043241707	3.22123E-17	1975	hypothetical protein BN946_scf184844.g12 [Trametes cinnabarina]
c25388_g2	0.107221388	7.50391E-17	4131	hypothetical protein DICSQDRAFT_155938 [Dichomitus squalens LYAD-421 SS1]
c19802_g1	0.054835408	8.16488E-17	2108	aminotransferase [Dichomitus squalens LYAD-421 SS1]
c24407_g2	0.018629692	9.76712E-17	1268	Zn-dependent exopeptidase [Dichomitus squalens LYAD-421 SS1]
c16113_g1	0.090138699	2.47608E-16	764	hypothetical protein BN946_scf184851.g102 [Trametes cinnabarina]
c14568_g1	0.012080273	1.76455E-15	888	Sec1-like protein [Dichomitus squalens LYAD-421 SS1]
c18638_g1	0.021965476	5.10562E-15	1325	-
c20516_g3	0.056383749	1.07085E-14	1927	hypothetical protein DICSQDRAFT_183608 [Dichomitus squalens LYAD-421 SS1]
c8700_g1	0.070399669	2.45128E-14	437	hypothetical protein DICSQDRAFT_155938 [Dichomitus squalens LYAD-421 SS1]
c24861_g1	0.103919719	2.71921E-14	2743	kinase-like protein [Trametes versicolor FP-101664 SS1]
c15848_g1	0.043479109	3.40658E-14	1218	hypothetical protein DICSQDRAFT_78106 [Dichomitus squalens LYAD-421 SS1]
c21255_g3	0.109150868	1.61513E-13	3096	cytochrome P450 [Dichomitus squalens LYAD-421 SS1]
c18585_g3	0.024500939	2.10115E-13	900	-
c23559_g2	0.086773613	4.089E-13	341	-
c17988_g1	0.013897139	1.01399E-12	432	-
c14426_g1	0.013185853	1.04158E-12	572	-
c3998_g1	0.004258895	1.4668E-12	858	-
c21705_g1	0.116561171	4.5242E-12	2429	hypothetical protein DICSQDRAFT_152375 [Dichomitus squalens LYAD-421 SS1]
c25232_g1	0.105728317	1.18501E-11	4842	hypothetical protein DICSQDRAFT_166043 [Dichomitus squalens LYAD-421 SS1]

c25293_g1	0.007113989	1.26307E-11	301	hypothetical protein BN946_scf184829.g34 [Trametes cinnabarina]
c24509_g2	0.117990511	1.63436E-11	3268	glycoside hydrolase [Dichomitus squalens LYAD-421 SS1]
c19435_g1	0.147916144	2.88909E-11	3381	hypothetical protein BN946_scf184884.g31 [Trametes cinnabarina]
c21141_g1	0.198378929	4.69153E-11	1989	hypothetical protein DICSQDRAFT_68969, partial [Dichomitus squalens LYAD-421 SS1]
c10175_g1	0.113953521	5.30475E-11	1854	hypothetical protein BN946_scf185007.g8 [Trametes cinnabarina]
c14964_g1	0.140556233	6.04359E-11	943	riboflavin kinase [Dichomitus squalens LYAD-421 SS1]
c11709_g1	0.018920061	7.21318E-11	579	-
c17123_g1	0.155177944	1.06452E-10	1964	zf-ZPR1-domain-containing protein [Dichomitus squalens LYAD-421 SS1]
c24638_g1	0.162312662	2.08022E-10	1937	hypothetical protein BN946_scf185043.g196 [Trametes cinnabarina]
c19576_g1	0.148546678	2.17813E-10	1565	hypothetical protein DICSQDRAFT_181999 [Dichomitus squalens LYAD-421 SS1]
c18617_g1	0.170448651	2.50538E-10	1946	hypothetical protein DICSQDRAFT_135488 [Dichomitus squalens LYAD-421 SS1]
c13303_g1	0.033309725	2.81967E-10	595	-
c24507_g1	0.130170508	4.28448E-10	3432	hypothetical protein DICSQDRAFT_55452 [Dichomitus squalens LYAD-421 SS1]
c24606_g1	0.061534591	4.97625E-10	1303	hypothetical protein DICSQDRAFT_112382 [Dichomitus squalens LYAD-421 SS1]
c17808_g1	0.177740882	4.98067E-10	1254	hypothetical protein TRAVEDRAFT_53328 [Trametes versicolor FP-101664 SS1]
c25654_g1	0.214185544	5.53205E-10	3182	P-loop containing nucleoside triphosphate hydrolase protein [Dichomitus squalens LYAD-421 SS1]
c21756_g2	0.010573232	5.7552E-10	1315	hypothetical protein DICSQDRAFT_174213 [Dichomitus squalens LYAD-421 SS1]
c23713_g1	0.151381373	6.04809E-10	6452	HET-domain-containing protein [Dichomitus squalens LYAD-421 SS1]
c24780_g4	0.17114564	7.98192E-10	3058	hypothetical protein DICSQDRAFT_161983 [Dichomitus squalens LYAD-421 SS1]
c19426_g1	0.114461103	8.11672E-10	1923	aromatic compound dioxygenase [Dichomitus squalens LYAD-421 SS1]

Table S6 GO enrichment between different samples (pvalue ≤ 0.05).

Catergory	GO_Term	Cluster frequency	Genome frequency of use	Corrected P-value
**GO enrichment between sample CK and HT**				
molecular_function	GO:0016641 oxidoreductase activity, acting on the CH-NH2 group of donors, oxygen as acceptor	3 out of 117 genes, 2.6%	11 out of 13372 genes, 0.1%	0.00951
**GO enrichment between sample HT and HTSNP**				
molecular function	GO:0051787 misfolded protein binding	4 out of 241 genes, 1.7%	5 out of 13372 genes, 0.0%	0.0000669
biological process	GO:0043335 protein unfolding	4 out of 241 genes, 1.7%	5 out of 13372 genes, 0.0%	0.00018
cellular component	GO:0034099 luminal surveillance complex	4 out of 241 genes, 1.7%	7 out of 13372 genes, 0.1%	0.0003
biological process	GO:0051084 'de novo' posttranslational protein folding	4 out of 241 genes, 1.7%	7 out of 13372 genes, 0.1%	0.00125
biological process	GO:0031204 posttranslational protein targeting to membrane, translocation	4 out of 241 genes, 1.7%	8 out of 13372 genes, 0.1%	0.00247
cellular component	GO:0005788 endoplasmic reticulum lumen	4 out of 241 genes, 1.7%	12 out of 13372 genes, 0.1%	0.00395
biological process	GO:0006620 posttranslational protein targeting to membrane	4 out of 241 genes, 1.7%	9 out of 13372 genes, 0.1%	0.00439
biological process	GO:0000742 karyogamy involved in conjugation with cellular fusion	4 out of 241 genes, 1.7%	9 out of 13372 genes, 0.1%	0.00439
biological process	GO:0000741 karyogamy	4 out of 241 genes, 1.7%	9 out of 13372 genes, 0.1%	0.00439
biological	GO:0042026 protein refolding	5 out of 241 genes,	18 out of 13372 genes,	0.00469

process		2.1%	0.1%	
biological process	GO:0006616 SRP-dependent cotranslational protein targeting to membrane, translocation	4 out of 241 genes, 1.7%	10 out of 13372 genes, 0.1%	0.00722
cellular component	GO:0031965 nuclear membrane	4 out of 241 genes, 1.7%	14 out of 13372 genes, 0.1%	0.00777
biological process	GO:0006986 response to unfolded protein	4 out of 241 genes, 1.7%	12 out of 13372 genes, 0.1%	0.01654
biological process	GO:0034605 cellular response to heat	5 out of 241 genes, 2.1%	23 out of 13372 genes, 0.2%	0.01713
biological process	GO:0035966 response to topologically incorrect protein	5 out of 241 genes, 2.1%	24 out of 13372 genes, 0.2%	0.02132
biological process	GO:0006458 'de novo' protein folding	4 out of 241 genes, 1.7%	14 out of 13372 genes, 0.1%	0.03252
molecular function	GO:0004144 diacylglycerol O-acyltransferase activity	2 out of 241 genes, 0.8%	2 out of 13372 genes, 0.0%	0.0427
**GO enrichment between sample CK and CKSNP**				
cellular_component	GO:0005840 ribosome	147 out of 1194 genes, 12.3%	569 out of 11962 genes, 4.8%	1.27E-26
molecular_function	GO:0003735 structural constituent of ribosome	128 out of 1194 genes, 10.7%	461 out of 11962 genes, 3.9%	6.31E-26
molecular_function	GO:0005198 structural molecule activity	132 out of 1194 genes, 11.1%	579 out of 11962 genes, 4.8%	3.89E-18
cellular_component	GO:0030529 intracellular ribonucleoprotein complex	171 out of 1194 genes, 14.3%	889 out of 11962 genes, 7.4%	5.28E-16
cellular_component	GO:1990904 ribonucleoprotein complex	171 out of 1194 genes, 14.3%	889 out of 11962 genes, 7.4%	5.28E-16

biological_process	GO:0043043 peptide biosynthetic process	155 out of 1194 genes, 13.0%	792 out of 11962 genes, 6.6%	2.2E-14
biological_process	GO:0006412 translation	154 out of 1194 genes, 12.9%	786 out of 11962 genes, 6.6%	2.54E-14
biological_process	GO:0006518 peptide metabolic process	158 out of 1194 genes, 13.2%	816 out of 11962 genes, 6.8%	2.91E-14
biological_process	GO:0043604 amide biosynthetic process	157 out of 1194 genes, 13.1%	820 out of 11962 genes, 6.9%	1.09E-13
biological_process	GO:0043603 cellular amide metabolic process	162 out of 1194 genes, 13.6%	865 out of 11962 genes, 7.2%	2.79E-13
cellular_component	GO:0044391 ribosomal subunit	60 out of 1194 genes, 5.0%	229 out of 11962 genes, 1.9%	2.58E-10
biological_process	GO:1901566 organonitrogen compound biosynthetic process	201 out of 1194 genes, 16.8%	1274 out of 11962 genes, 10.7%	4.1E-09
cellular_component	GO:0022626 cytosolic ribosome	44 out of 1194 genes, 3.7%	151 out of 11962 genes, 1.3%	6.15E-09
cellular_component	GO:0043228 non-membrane-bounded organelle	186 out of 1194 genes, 15.6%	1217 out of 11962 genes, 10.2%	0.000000109
cellular_component	GO:0043232 intracellular non-membrane-bounded organelle	186 out of 1194 genes, 15.6%	1217 out of 11962 genes, 10.2%	0.000000109
biological_process	GO:1901564 organonitrogen compound metabolic process	234 out of 1194 genes, 19.6%	1598 out of 11962 genes, 13.4%	0.000000118
biological_process	GO:0044271 cellular nitrogen compound biosynthetic process	197 out of 1194 genes, 16.5%	1296 out of 11962 genes, 10.8%	0.000000209
cellular_component	GO:0022627 cytosolic small ribosomal subunit	23 out of 1194 genes, 1.9%	61 out of 11962 genes, 0.5%	0.00000149

biological_process	GO:0034645 cellular macromolecule biosynthetic process	172 out of 1194 genes, 14.4%	1122 out of 11962 genes, 9.4%	0.00000191
biological_process	GO:0009059 macromolecule biosynthetic process	173 out of 1194 genes, 14.5%	1138 out of 11962 genes, 9.5%	0.00000324
cellular_component	GO:0015935 small ribosomal subunit	30 out of 1194 genes, 2.5%	100 out of 11962 genes, 0.8%	0.00000413
biological_process	GO:1901576 organic substance biosynthetic process	244 out of 1194 genes, 20.4%	1787 out of 11962 genes, 14.9%	0.0000368
biological_process	GO:0044249 cellular biosynthetic process	236 out of 1194 genes, 19.8%	1740 out of 11962 genes, 14.5%	0.00011
biological_process	GO:0009058 biosynthetic process	249 out of 1194 genes, 20.9%	1875 out of 11962 genes, 15.7%	0.00029
cellular_component	GO:0044445 cytosolic part	48 out of 1194 genes, 4.0%	243 out of 11962 genes, 2.0%	0.00058
cellular_component	GO:0015934 large ribosomal subunit	30 out of 1194 genes, 2.5%	129 out of 11962 genes, 1.1%	0.00162
biological_process	GO:0019538 protein metabolic process	245 out of 1194 genes, 20.5%	1932 out of 11962 genes, 16.2%	0.01425
biological_process	GO:0006414 translational elongation	20 out of 1194 genes, 1.7%	73 out of 11962 genes, 0.6%	0.01855
biological_process	GO:0010467 gene expression	186 out of 1194 genes, 15.6%	1409 out of 11962 genes, 11.8%	0.01892
biological_process	GO:0044267 cellular protein metabolic process	217 out of 1194 genes, 18.2%	1689 out of 11962 genes, 14.1%	0.02131
cellular_component	GO:0005737 cytoplasm	366 out of 1194 genes, 30.7%	3122 out of 11962 genes, 26.1%	0.02383

cellular_component	GO:0044444 cytoplasmic part	309 out of 1194 genes, 25.9%	2585 out of 11962 genes, 21.6%	0.02526
cellular_component	GO:0022625 cytosolic large ribosomal subunit	19 out of 1194 genes, 1.6%	79 out of 11962 genes, 0.7%	0.04601

Table S7 KEGG enrichment between different samples (pvalue ≤ 0.05).

Pathway_ID	Pathway	level1	level2	pvalue	FDR
**KEGG enrichment between sample CK and HT**					
ko00604	Glycosphingolipid biosynthesis - ganglio series	Metabolism	Glycan biosynthesis and metabolism	0.01033295	0.26328848
ko00603	Glycosphingolipid biosynthesis - globo series	Metabolism	Glycan biosynthesis and metabolism	0.01033295	0.26328848
ko00531	Glycosaminoglycan degradation	Metabolism	Glycan biosynthesis and metabolism	0.02056305	0.26328848
ko04141	Protein processing in endoplasmic reticulum	Genetic Information Processing	Folding, sorting and degradation	0.02276099	0.26328848
ko04621	NOD-like receptor signaling pathway	Organismal Systems	Immune system	0.0407186	0.26328848
ko00643	Styrene degradation	Metabolism	Xenobiotics biodegradation and metabolism	0.0407186	0.26328848
ko04612	Antigen processing and presentation	Organismal Systems	Immune system	0.0407186	0.26328848
ko00520	Amino sugar and nucleotide sugar metabolism	Metabolism	Carbohydrate metabolism	0.04460045	0.26328848
**KEGG enrichment between sample HT and HTSNP**					
ko04214	Apoptosis - fly	Cellular Processes	Cell growth and death	0.01602328	0.17822038
ko00071	Fatty acid degradation	Metabolism	Lipid metabolism	0.02115784	0.17822038
ko04212	Longevity regulating pathway - worm	Organismal Systems	Aging	0.0268820	0.1782203

				8	8
ko00040	Pentose and glucuronate interconversions	Metabolism	Carbohydrate metabolism	0.02995316	0.17822038
ko00592	alpha-Linolenic acid metabolism	Metabolism	Lipid metabolism	0.03038758	0.17822038
ko04215	Apoptosis - multiple species	Cellular Processes	Cell growth and death	0.03038758	0.17822038
ko00380	Tryptophan metabolism	Metabolism	Amino acid metabolism	0.03315728	0.17822038
ko04213	Longevity regulating pathway - multiple species	Organismal Systems	Aging	0.03315728	0.17822038
**KEGG enrichment between sample CK and CKSNP**					
ko03010	Ribosome	Genetic Information Processing	Translation	3.47078E-19	6.6986E-17
ko04626	Plant-pathogen interaction	Organismal Systems	Environmental adaptation	7.99721E-05	0.007228242
ko00100	Steroid biosynthesis	Metabolism	Lipid metabolism	0.000112356	0.007228242
ko01200	Carbon metabolism	Metabolism	Overview	0.000241978	0.011675419
ko00710	Carbon fixation in photosynthetic organisms	Metabolism	Energy metabolism	0.001518554	0.058616184
ko04612	Antigen processing and presentation	Organismal Systems	Immune system	0.002869846	0.079653637
ko00630	Glyoxylate and dicarboxylate metabolism	Metabolism	Carbohydrate metabolism	0.002888992	0.079653637

ko04212	Longevity regulating pathway - worm	Organismal Systems	Aging	0.006391946	0.147447386
ko00053	Ascorbate and aldarate metabolism	Metabolism	Carbohydrate metabolism	0.00704118	0.147447386
ko04728	Dopaminergic synapse	Organismal Systems	Nervous system	0.007906173	0.147447386
ko04152	AMPK signaling pathway	Environmental Information Processing	Signal transduction	0.008403737	0.147447386
ko00010	Glycolysis / Gluconeogenesis	Metabolism	Carbohydrate metabolism	0.01209819	0.173420729
ko03320	PPAR signaling pathway	Organismal Systems	Endocrine system	0.01319791	0.173420729
ko00791	Atrazine degradation	Metabolism	Xenobiotics biodegradation and metabolism	0.01540401	0.173420729
ko04744	Phototransduction	Organismal Systems	Sensory system	0.01570353	0.173420729
ko04745	Phototransduction - fly	Organismal Systems	Sensory system	0.01570353	0.173420729
ko04011	MAPK signaling pathway - yeast	Environmental Information Processing	Signal transduction	0.01732358	0.173420729
ko00340	Histidine metabolism	Metabolism	Amino acid metabolism	0.01777165	0.173420729
ko04261	Adrenergic signaling in cardiomyocytes	Organismal Systems	Circulatory system	0.01777165	0.173420729
ko00680	Methane metabolism	Metabolism	Energy metabolism	0.01797106	0.173420729

ko00330	Arginine and proline metabolism	Metabolism	Amino acid metabolism	0.02606397	0.234317966
ko04750	Inflammatory mediator regulation of TRP channels	Organismal Systems	Sensory system	0.02670982	0.234317966
ko04621	NOD-like receptor signaling pathway	Organismal Systems	Immune system	0.02853722	0.239464498
ko02020	Two-component system	Environmental Information Processing	Signal transduction	0.0299326	0.240707992
ko04915	Estrogen signaling pathway	Organismal Systems	Endocrine system	0.03936114	0.289792177
ko00040	Pentose and glucuronate interconversions	Metabolism	Carbohydrate metabolism	0.04207826	0.289792177
ko00051	Fructose and mannose metabolism	Metabolism	Carbohydrate metabolism	0.04207826	0.289792177
ko00620	Pyruvate metabolism	Metabolism	Carbohydrate metabolism	0.04535261	0.289792177
ko04740	Olfactory transduction	Organismal Systems	Sensory system	0.04540866	0.289792177
ko04970	Salivary secretion	Organismal Systems	Digestive system	0.04540866	0.289792177
ko04922	Glucagon signaling pathway	Organismal Systems	Endocrine system	0.04654693	0.289792177

Table S8 Unigenes used for validation of gene expression profile in *G. oregonense* transcriptome data.

**Group**	**Gene ID**	**Differential expression analysis based on RNA-seq**		**Validation of the DEG by RT-PCR analysis**				**Accession NO.**
		log2Fold Change	pvalue	CK (2-⊿⊿Ct)	HT (2-⊿⊿Ct)	HTSNP (2-⊿⊿Ct)	Pvalue	
CK VS HT	DN19739_c0_g3	3.34	3.26E-10	1.00±0.01	0.34±0.00	-	3.52E-05	KY781319
	DN26323_c1_g4	2.36	2.29E-06	2.69±0.15	1.00±0.01	-	1.92E-03	KY781320
	DN26299_c2_g4	3.46	5.53E-06	1.01±0.02	0.10±0.00	-	2.57E-04	KY781321
HTSNP VS HT	DN26105_c1_g1	3.03	4.53E-06	-	0.51±0.04	1.29±0.01	4.19E-04	KY781322
	DN18358_c0_g1	2.57	1.83E-05	-	1.01±0.02	1.54±0.01	7.01E-03	KY781323
	DN25853_c0_g5	8.44	3.42E-05	-	1.00±0.00	55.76±25.76	4.83E-05	KY781324
CK VS HTSNP	DN26608_c3_g1	-2.3	8.53E-06	1.00±0.00	-	4.63±0.06	1.60E-05	KY781325
	DN21758_c0_g2	-5.54	8.53E-06	1.00±0.01	-	3.11±0.07	1.98E-04	KY781326
	DN39269_c0_g1	-2.92	8.53E-06	1.00±0.00	-	5.85±0.12	1.76E-05	KY781327

2-⊿⊿Ct, relative gene expression level with RPL4 serving as the reference gene using RT-PCR analysis. Data are presented as means ±standard deviation (SD) of three replicates. CK, *G. oregonense* mycelia treated at 28 ° C; HT, *G. oregonense* mycelia treated at 32 ℃; HTSNP, *G. oregonense* mycelia treated at 32 ℃ with 100 μM SNP.

Table S9 Primers designed for validation of gene expression profile in *G. oregonense* transcriptome data.

Gene ID	Primer ID	Primer sequences	Amplified products
DN19739_c0_g3	CC1-F	CTCCCTCCAACCGCATAGAT	107 bp
	CC1-R	GACCTCCTTTTCCCCGCTAA	
DN26323_c1_g4	CC2-F	TACTGGAAGGGAGCCAACCA	149 bp
	CC2-R	TTTCTCGGTGCGTACTCTCG	
DN26299_c2_g4	CC3-F	AGCACCCACATCCTCTGTATTC	114 bp
	CC3-R	GGGAGACAGGTCAGAAGACATT	
DN26105_c1_g1	CC4-F	GTTTGGCCGATGTGGAATGG	203 bp
	CC4-R	GCCGTGGTGCCTATACTCTC	
DN18358_c0_g1	CC5-F	GGAGAATCCACTCGCTCGAC	220 bp
	CC5-R	AATGTACCCCGCAGTAGCAC	
DN25853_c0_g5	CC6-F	TCGTGTCGTGGAGAACAAGG	113 bp
	CC6-R	CCTTCTTGGGCTTCGCAAAC	
DN26608_c3_g1	CC7-F	CAGGATGTCTCGTCCGATGG	117 bp
	CC7-R	GGCGAATGACGAGAGAGAGG	
DN21758_c0_g2	CC8-1F	GTCCCGACACGTCTGTTCTT	109 bp
	CC8-1R	GAGAGCCGATAGCGTCCAAA	
DN39269_c0_g1	CC9-F	CCATCAACGAGGCTACGGTT	101 bp
	CC9-R	GCCATCGGACAAGACATCCT	
RPL4(reference gene)	RPL4-F	GTCAACAAGGGCGTTCTCTT	151 bp
	RPL4-R	ACAGCGTCTTGAGGAAGGT	
